# Efficient Desulfurizer Recycling during Spent Lead–Acid Batteries Paste Disposal by Zero‐Carbon Precursor Hypothermic Smelting

**DOI:** 10.1002/advs.202405168

**Published:** 2024-09-20

**Authors:** Fei Li, Neng‐Wu Zhu, Yun‐Hao Xi, Wu‐Wan Xiong, Ju‐Jun Ruan, Xiao‐Rong Wei, An‐Qi Guo, Yi‐Jun Chen, Ping‐Xiao Wu, Zhi Dang

**Affiliations:** ^1^ School of Environment and Energy South China University of Technology Guangzhou 510006 China; ^2^ School of Environmental Science and Engineering Sun Yat‐Sen University Guangzhou 510275 China; ^3^ The Key Lab of Pollution Control and Ecosystem Restoration in Industry Cluster Ministry of Education South China University of Technology Guangzhou 510006 China; ^4^ Guangdong Environmental Protection Key Laboratory of Solid Waste Treatment and Recycling South China University of Technology Guangzhou 510006 China; ^5^ Guangdong Provincial Key Laboratory of Solid Wastes Pollution Control and Recycling South China University of Technology Guangzhou 510006 China

**Keywords:** desulfurizer recycling, hypothermic smelting, sodium molybdate, spent lead–acid batteries, zero‐carbon precursor

## Abstract

Recycling of spent lead‐acid batteries (LABs) is extremely urgent in view of environmental protection and resources reuse. The current challenge is to reduce high consumption of chemical reagents. Herein, a closed‐loop spent LABs paste (SLBP) recovery strategy is demonstrated through Na_2_MoO_4_ consumption‐regeneration‐reuse. Experimental and DFT calculations verify that MoO_4_^2−^ competes Pb/Ca ions and weakens the metal‐oxygen bond of PbSO_4_/CaSO_4_.2H_2_O in SLBP, facilitating PbMoO_4_/CaMoO_4_ formation and 99.13 wt% of SO_4_^2−^ elimination. Pb of 99.97 wt% is obtained as zero‐carbon precursors (PbO_2_ and PbMoO_4_) by green leaching coupled with re‐crystallization. The regeneration of Na_2_MoO_4_ is realized at 600 ℃ using LABs polypropylene shells and NaOH as reagents. Compared with the traditional smelting technologies, the temperature is reduced from ＞1000 to 600 °C. The extraction of Na_2_MoO_4_ require only water, and satisfactory re‐used desulfurization efficiency (98.67 wt%) is achieved. For the residual Na_2_MoO_4_ after first SLBP desulfurization, the desulfurization efficiency remains above 97.36 wt% after adding fresh reagents for two running cycles. The new principle enables the reuse of 99.83 wt% of Na_2_MoO_4_ and the recycling of 95.27 wt% of Pb without generating wastewater and slags. The techno‐economic analysis indicates this strategy is efficient, economical, and environmentally‐friendly.

## Graphical abstract

For lead recovery from spent LABs paste, chemical reagents consumption, lead yield, and environmental risk are crucial factors for the low‐cost sustainable development of recycled lead enterprises. Herein, a closed‐loop lead recovery strategy is demonstrated through Na_2_MoO_4_ consumption–regeneration–reuse. Noteworthily, 99.83 wt% of Na_2_MoO_4_ is reused and 95.27 wt% of Pb is recycled without generating wastewater and slags.

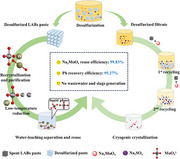

## 1 Introduction

With the rapid development of the automobile industry, the production of lead–acid batteries (LABs) as the automotive ignition power source and energy storage devices has experienced enormous growth during the past few decades.^[^ ^1^ ^]^ Up to 11.7 million tons of refined lead (Pb) were used in the manufacture of LABs, accounting for over 85% of the total global refined Pb production in 2020.^[^ ^2^ ^]^ The lifecycle of current LABs is typically around 2–5 years due to the internal deterioration of battery.^[^ ^3^ ^]^ The large‐scale use of LABs inevitably produced huge quantities of spent LABs.^[^ ^4^ ^]^ Relevant studies reported that the annual amount of spent and discarded LABs was more than 2.6 million tons in China, ≈1.8 million tons in the Americas, and ≈1.5 million tons in Europe.^[^ ^5^ ^]^ With the exhaustion of primary Pb resources, the spent LABs have emerged as the most significant secondary Pb resource.^[^ ^4a^ ^]^ Developing the secondary Pb industry to create a virtuous Pb cycle of production–consumption–regeneration will not only alleviate the depletion of Pb resources, but also reduce production costs and environmental pollution, which is of strategic significance in promoting the sustainable development of global Pb industry.

Spent LABs paste (SLBP), formed by the active material after long‐term charge and discharge, is the high‐quality lead‐containing resource recovered from spent LABs, which mainly contains Pb(II)SO_4_ (≈60%), Pb(IV)O_2_ (≈30%), Pb(II)O (≈9%), Pb (≈1%), and other impurities components (1%) such as Fe, Ba, Al, Sn, and Sb.^[^ ^6^ ^]^ Among the components of SLBP, Pb(II)SO_4_ is the main lead compound and is very difficult to dispose of due to its thermodynamic stability, even under a high temperature of 1300 °C. The decomposition of Pb(II)SO_4_ phases leads to the emission of lead particulates (30–50 kg t^−1^), sulfur dioxide (≈70 kg t^−1^), and requires a lot of energy.^[^ ^7^ ^]^ With increasingly stringent legislation on pyrometallurgical emission standards, there has been a gradual shift in research focus to desulfurization‐based recovery processes, including carbonate/alkaline desulfurization followed by recrystallization^[^ ^8^ ^]^ and organic acid desulfurization followed by calcination process.^[^ ^1^, ^6^, ^9^ ^]^ Up to now, carbonate (K_2_CO_3_, Na_2_CO_3_, and (NH_4_)_2_CO_3_), alkali (NaOH), and organic acids (citric acid/sodium citrate, acetic acid/sodium citrate_,_ acetic acid, and oxalate/sodium oxalate have been developed as desulfurization reagents for SLBP to achieve the conversion of high‐stability Pb(II)SO_4_ to lower decomposition temperatures of lead compounds (i.e., lead carbonate, lead oxalate, and lead citrate).^[^ ^1c^ ^]^ More importantly, the above desulfurization‐based recovery technologies lay the foundation for efficient recovery of Pb while controlling SO_2_ gas and Pb vapor/dust pollution.

In light of carbon peaking by 2030 and carbon neutrality by 2060 goals, researchers are no longer just satisfied with efficient metal recovery rate but are also beginning to care about how to reduce the consumption of desulfurization reagents in the recovery process to minimize the environmental footprint while enhancing profitability. This is primarily attributed to the following limitations of the reported desulfurizers: 1) to achieve high desulfurization efficiency, the chemical reagents are often used in excess, resulting in a substantial amount of waste chemicals and potential environmental risks;^[^ ^10^ ^]^ 2) the use of expensive non‐recyclable reagents significantly increased the cost of Pb recovery technology, hindering its industrial applications;^[^ ^11^ ^]^ and 3) the high CO_2_ emissions during subsequent smelting process of high carbon‐content smelting precursors (i.e., lead carbonate, lead oxalate, and lead citrate) were also unacceptable for modern industry. Till now, desulfurization filtrate re‐circulation and desulfurizers reuse in the spent SLBP recovery process have not been reported in previous studies. Therefore, the development of desulfurizers that balance high desulfurization efficiency, recyclability, carbon free, and economical is crucial for the sustainable development of reclaimed Pb industry.

Herein, we propose a closed‐loop SLBP recovery strategy through Na_2_MoO_4_ consumption–regeneration–reuse. Na_2_MoO_4_ is a widely used environmental remediation, has the solubility product (Ksp(PbMoO_4_) = 1.0 × 10^−13^) of its lead salt (PbMoO_4_) is five orders of magnitude lower than that of PbSO_4_(Ksp(PbSO_4_) = 1.6 × 10^−8^) (“Lange's Handbook of Chemistry”). The behavior of Na_2_MoO_4_ on SLBP, recrystallisation and purification of desulfurized SLBP (DLBP), regeneration mechanism of Na_2_MoO_4_ and its reuse efficiency feasibility and effectiveness were studied by X‐ray diffraction, Fourier transformed infrared, field emission scanning electron microscopy, inductively coupled plasma optical emission spectroscopy, anion chromatography, and thermodynamics calculations, and DFT calculations. Using an integrated multi‐step process, we recycled SLBP as PbO with a high Pb recycling yield of 95.27 and 99.83 wt% Na_2_MoO_4_ could be regenerated/recovered for SLBP desulfurization without waste liquid and slags. Compared with the traditional smelting technology, the temperature was reduced from >1000 to 600 °C, thus reducing the energy consumption. The desulfurization performance (above 95.34 wt%) of recovered Na_2_MoO_4_ was comparable to that of fresh Na_2_MoO_4_, which confirmed the feasibility of Na_2_MoO_4_ reuse.

## 2 Results and Discussion

### 2.1 Desulfurization Behavior of Na_2_MoO_4_ and Preparation of Zero‐Carbon Precursor

**Figure 1:**
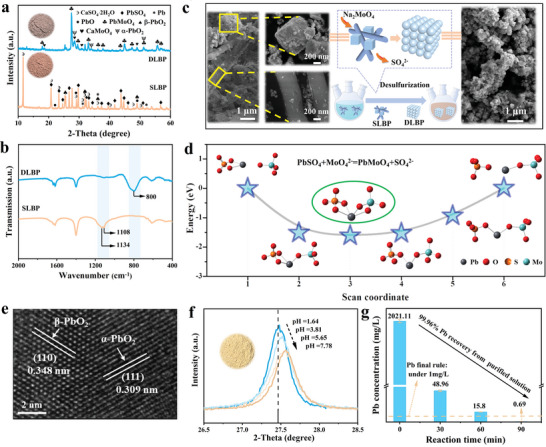
a) XRD spectrum of SLBP before reaction and after reaction (S/S = 2:1, L/S = 5:1, *T* = 90 °C and *t* = 4 h). b) FTIR spectra of SLBP and DLBP. c) Reaction flow chart of SLBP desulfurization. d) Energetic and structural changes in the removal of SO_4_^2−^ from PbSO_4_ in the presence of Na_2_MoO_4_. e) HRTEM image of residues after HNO_3_ leaching. f) Enlarged XRD pattern of re‐crystallization product of HNO_3_ purified solution at different pH values (1.64, 3.81, 5.65, and 7.78). g) Recovery of Pb in HNO_3_ purification solution by NaOH.

The phase analysis of the ≤74 µm crushed product of SLBP is illustrated in **Figure** **1**a. The product consists a mixture of PbSO_4_, β‐PbO_2_, PbO, Pb, and CaSO_4_^.^2H_2_O, which aligns well with the reported data.^[^ ^12^ ^]^ The theoretical feasibility of SLBP desulfurization was evaluated from solubility constants and thermodynamics according to the “Lange's Handbook of Chemistry”. The change in solubility product for the conversion of PbSO_4_ (Ksp(PbSO_4_) = 1.6 × 10^−8^) to PbMoO_4_ (Ksp(PbMoO_4_) = 1.0 × 10^−13^) and CaSO_4_^.^2H_2_O (Ksp(CaSO_4_^.^2H_2_O) = 1 × 10^−4.5^) to CaMoO_4_ (Ksp(CaMoO_4_) = 1.46 × 10^−8^) is five and three orders of magnitude, respectively, and the Gibbs free energy values (Δ*G*) are both negative, indicating that the desulfurization process is spontaneous (Figure S1, Supporting Information). To gain further insight into the desulfurization efficiency of SLBP in the presence of Na_2_MoO_4_, the influence of various process parameters, that is, Na_2_MoO_4_/SLBP mass ratio (S/S), liquid–solid ratio (L/S), temperature (*T*), and reaction time (*t*) on the treatment of SLBP were further studied. Compared with the XRD pattern of original SLBP, the characteristic peak of PbSO_4_ decreased greatly in the Na_2_MoO_4_ desulfurization system, and the characteristic peak of PbSO_4_ and CaSO_4_^.^2H_2_O even disappeared, corresponding to the formation of PbMoO_4_ and CaMoO_4_, respectively (Figure S2, Supporting Information). Among them, the characteristic peaks of CaMoO_4_, PbO, and α‐PbO_2_ overlapped. When the S/S ratio increased to 2:1, PbSO_4_ further generated PbMoO_4_. However, little variation could be observed when S/S was higher than 2:1, which might be because the desulfurized product layer of PbMoO_4_ hindered the mass transfer of the desulfurizer.^[^ ^10^ ^]^ The intensity of the PbSO_4_ characteristic peak increased with the increasing L/S (Figure S3, Supporting Information), indicating that the concentration of Na_2_MoO_4_ played an important role in the phase transition of PbSO_4_.^[^ ^13^ ^]^ With the temperature elevated, the intensity of the PbSO_4_ characteristic peak decreased gradually and disappeared at 90 °C (Figure S4, Supporting Information). It might be attributed to the enhanced activity of the reaction between Na_2_MoO_4_ and PbSO_4_ at high temperatures, which increased the disintegration rate of PbSO_4_.^[^ ^10^ ^]^ PbSO_4_ was gradually consumed with the increase of *t* until the desulfurization reaction was complete after 4 h (Figure S5, Supporting Information).

FTIR measurements of initial SLBP and DLBP were further performed (Figure 1b). The absorption bands at 1134 and 1108 cm^−1^ correspond to SO_4_^2−^ of PbSO_4_ and CaSO_4_·2H_2_O in SLBP.^[^ ^14^ ^]^ Different from the original SLBP, the characteristic peak of SO_4_^2−^ disappeared and the characteristic peak of Mo─O (800 cm^−1^) appeared in DLBP.^[^ ^15^ ^]^ According to the elemental mapping map, Mo could be detected in DLBP obtained after a 4‐h treatment of SLBP with Na_2_MoO_4_, while S could not be detected (Figure S6, Supporting Information). The above results demonstrated that desulfurization of SLBP could be successfully achieved through the reaction with Na_2_MoO_4_. The contents of the main elements in DLBP were determined by ICP‐OES (Table S1, Supporting Information). Compared with the original SLBP, the S content in DLBP decreased sharply from 100 563.2 to 1355.4 mg kg^−1^, and the desulfurization efficiency was calculated to be 99.13 wt% according to Equation S1 (Supporting Information). The SLBP desulfurization processes are shown in Figure 1c. In the presence of Na_2_MoO_4_, the raw SLBP, which assembled cubic and tubular structures, was converted into the homogeneous particulate matter while releasing SO_4_^2−^ (Figure 1a; Figure S7 and Table S2, Supporting Information).

To clarify the removal mechanism of SO_4_^2−^ from SLBP by MoO_4_^2−^, the distance between MoO_4_^2−^ ions and SO_4_^2−^ ions of PbSO_4_/CaSO_4_·2H_2_O was scanned, starting from a farther distance, so that the distance was gradually decreased until the most stable state, and then the distance was scanned with the increase of distance to simulate the process by which MoO_4_^2−^ competed for Pb or Ca of PbSO_4_/CaSO_4_·2H_2_O and released SO_4_^2−^. As MoO_4_^2−^ kept approaching PbSO_4_ from a distance, the number of Mo─O─Pb ionic bonds would change from one to two, at which point the coordination form between Pb ions and MoO_4_^2−^ as well as SO_4_^2−^ reached the most stable structure, and this process occurred spontaneously, with reduced energy. During the increasing distance, one Mo─O─Pb and S─O─Pb ionic bond was broken each, which in turn further broke the S─O─Pb ionic bond to form PbMoO_4_ and release SO_4_^2−^ (Figure 1d). The Ca ions changed in much the same way as the Pb ions. In the process of increasing distance, Ca ions were deflected toward MoO_4_^2−^, and as MoO_4_^2−^ gradually moved away from SO_4_^2−^, the ionic bond of Ca─O─S changed from two to one, and then until it broke, finally forming CaMoO_4_ (Figure S8, Supporting Information).

Nitric acid (HNO_3_) is used for the removal of impurities in DLBP to improve the grade of the lead compound. The degree of attenuation of CaMoO_4_, PbO, and PbMoO_4_ enhanced significantly with increasing HNO_3_/H_2_O volume ratio (Figure S9, Supporting Information). Under the conditions of HNO_3_/H_2_O = 17.5:62.5 and *t* = 2 h, the complex form in DLBP was transformed into a simpler form of the mixture of α‐PbO_2_ and β‐PbO_2_) (Figures S10 and S11, Supporting Information). In the HRTEM image of the residues after HNO_3_ leaching in Figure 1e, the lattice fringe spacing of 0.348 and 0.309 nm corresponded to the (110) crystal plane of β‐PbO_2_ and the (111) crystal plane of α‐PbO_2_, respectively. The corresponding EDS and elemental mapping analysis showed that Pb and O were uniformly distributed in the residues after HNO_3_ leaching (Figures S12 and S13, Supporting Information). As suggested by AAS results, the purity of recovered PbO_2_ reached 93.67%.

The types and contents of the main components in the acid‐purified liquid of DLBP are shown in Table S3 (Supporting Information). As the molar amount of Mo ions was slightly greater than that of Pb ions, the recovery of Pb could theoretically be achieved through the formation of PbMoO_4_. NaOH solution was used to adjust the pH of the HNO_3_ purification solution for the recovery of Pb. The XRD results showed that Pb in HNO_3_ purification solution all re‐crystallized in the form of PbMoO_4_ at different pH conditions (1.64, 3.81, 5.65, and 7.78) (Figure S14, Supporting Information). As shown in the enlarged XRD pattern (Figure 1f), the diffraction degree of PbMoO_4_ increased from 26.50° to 28.50° and the characteristic peaks were shifted to high angles with the increasing pH value. It might be due to the involvement of other cations in the re‐crystallization process of PbMoO_4_ which negatively affected the formation of PbMoO_4_. Afterward, the reaction time was increased to achieve the maximum recovery of Pb and Mo under the condition of pH = 1.64. The results showed that the concentration of Pb in the acid‐purified solution gradually decreased (Figure S15 and Table S4, Supporting Information). According to Equation S2 (Supporting Information), the maximum recovery efficiency of Pb reached 99.96 wt% (Figure 1g), and the content of Pb (0.69 mg L^−1^) in the final filtrate was lower than the integrated wastewater discharge standard of the People's Republic of China (GB8978‐1996). PbMoO_4_ obtained under the optimal condition was particles with an average size of 49.8 nm, and the main elements of it were Pb, Mo, and O which were uniformly distributed (Figures S16–S18, Supporting Information). The purity of recovered PbMoO_4_ reached 98.34%, as tested by ICP‐OES. Noteworthy, the recovery efficiency of Pb was 99.97 wt% and was present in the form of PbMoO_4_ and PbO_2_ (zero‐carbon precursors) in the whole purification and pH adjustment process according to the Equation S3 (Supporting Information).

### 2.2 Hypothermic Smelting of Zero‐Carbon Precursor and Water‐Leaching Extraction of Na_2_MoO_4_

**Figure 2:**
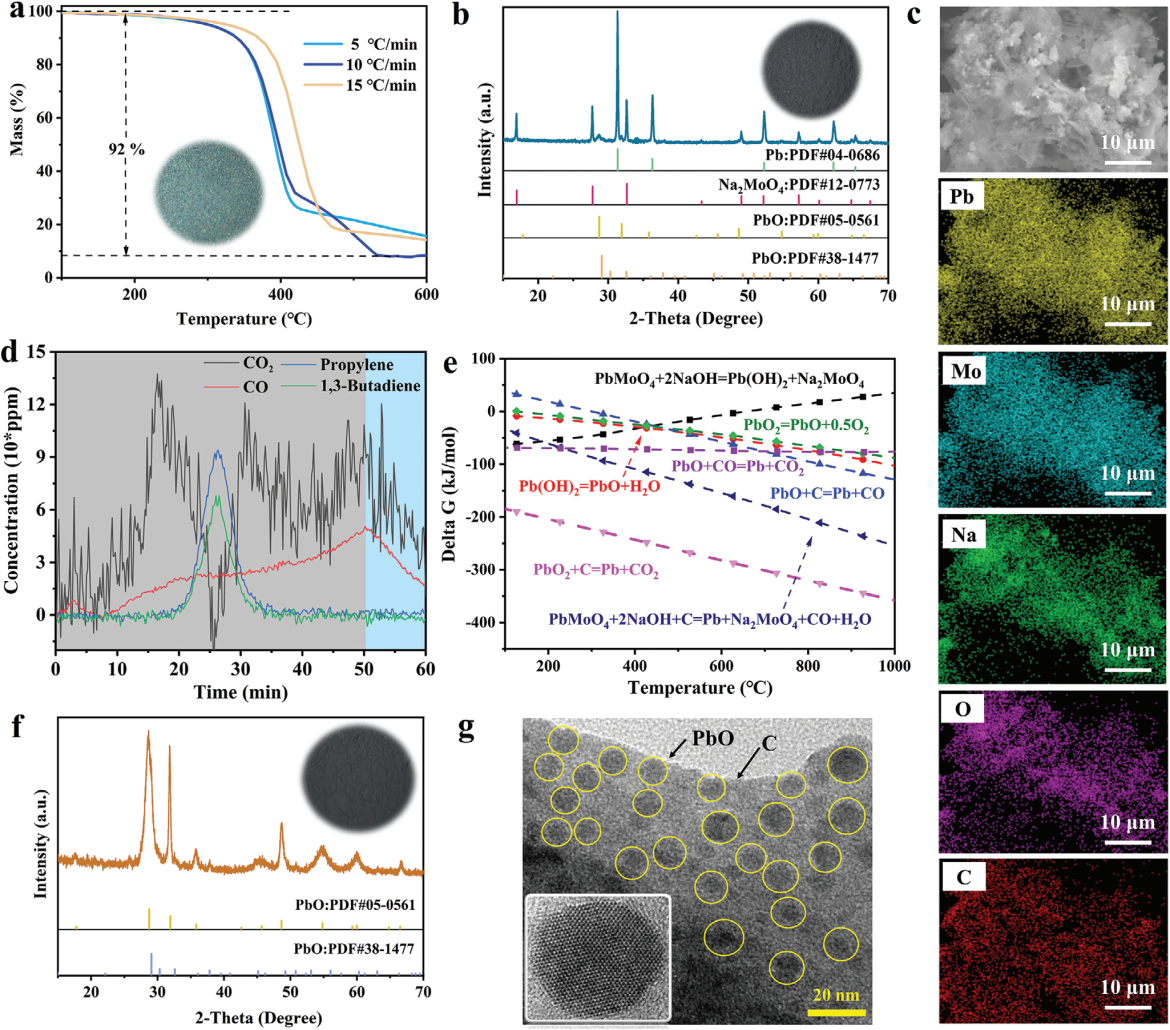
a) TG curves of the LABs polypropylene shells at different heating rates (5, 10, and 15 °C min^−1^). b) XRD patterns of smelting precursors (PbMoO_4_ and PbO_2_) after optimal reaction conditions (PbMoO_4_:PbO_2_:LABs polypropylene shells: NaOH = 4.38:0.66:2.78:1.05, *T* = 600 °C, and *t* = 12 h). c) Element mapping images of smelting precursors (PbMoO_4_ and PbO_2_) after optimal reaction conditions (PbMoO_4_:PbO_2_:LABs polypropylene shells: NaOH = 4.38:0.66:2.78:1.05, *T* = 600 °C, and *t* = 12 h). d) Composition and variation law of gas‐phase of smelting precursors (PbMoO_4_ and PbO_2_) after optimal reaction conditions (PbMoO_4_:PbO_2_:LABs polypropylene shells: NaOH = 4.38:0.66:2.78:1.05, *T* = 600 °C, and *t* = 12 h). e) Δ*G* values of possible thermodynamic reactions during the smelting reduction process of smelting precursors. f) XRD patterns of water‐leaching residues. g) HRTEM images of water‐leaching residues.

Low‐value LAB polypropylene shells were used as a substitute for reducing agents such as zero‐valent iron and biochar. The thermogravimetric curves of LAB polypropylene shells were first studied at different heating rates. As shown in **Figure** **2**a, the carbon mass produced and the weight loss exhibited similar trends when the heating rate was within the range of 5–15 °C min^−1^, at 8% and 92% respectively. Considering both time and energy costs, 10 °C min^−1^ was chosen as the optimum heating rate in this process. Further, the effects of different parameters (i.e., smelting temperature (*T*), reaction time (*t*), and the mass of LABs polypropylene shells) on the recovery efficiency of Pb and Mo were studied using the reaction equipment shown in Figure S19, Supporting Information. The XRD patterns of the zero‐carbon precursors reduction under dynamic N_2_ atmosphere at temperatures from 400 to 800 °C for 6 h (Figure S20, Supporting Information). Zero‐carbon precursors were converted to Na_2_MoO_4_, Pb^0^, and PbO at 400 °C using LABs polypropylene shells and NaOH as the carbon and sodium sources, respectively. In order to further promote the conversion of PbO to metallic Pb, higher smelting temperatures were studied. When the smelting temperature increased to 700 °C, the Mo_2_C characteristic peak began to appear,^[^ ^16^ ^]^ which would affect the subsequent Mo recovery efficiency. Considering the recovery capability of Na_2_MoO_4_ and energy efficiency, and optimal smelting temperature was determined as 600 °C. When *t* was increased to 12 h, PbO was further converted to Pb (Figure S21, Supporting Information). However, little change was observed when *t* exceeded 12 h, which could be explained by the fact that the content of carbon was insufficient for the further reduction of PbO. When the mass of LABs polypropylene shells was increased from 2.29 to 2.79 g, the characteristic peak of PbO decreased gradually, but a further increase of the mass of LABs polypropylene shells did not occur in further reduction of PbO (Figure S22, Supporting Information). The incomplete transformation of PbO with a large number of LABs polypropylene shells might be due to the negative influence of excessive carbon on the thermal conductivity process. In short, the optimal condition for zero‐carbon precursors converted to Na_2_MoO_4_, Pb, and PbO was determined to PbMoO_4_:PbO_2_:LABs polypropylene shells: NaOH = 4.38:0.66:2.79: 1.05, *T* = 600 °C, and *t* = 12 h (Figure 2b). Compared with the traditional smelting technology, the temperature was reduced from >1000 to 600 °C, thus reducing the energy consumption. In the HRTEM image of the smelting products in Figure S23 (Supporting Information), the lattice fringe spacing of 0.283 and 0.272 nm corresponds to the (111) crystal plane of Pb and the (311) crystal plane of Na_2_MoO_4_, respectively. In addition, the element mapping images showed that the main elements were Pb, Mo, Na, C, and O, which were highly consistent with the XRD results (Figure 2c). The recovery efficiency of Pb and Mo were to be 95.27 and 100 wt%, respectively, as determined by ICP‐OES and Equation S4 (Supporting Information).

To further deepen the understanding of the hypothermic smelting process of zero‐carbon precursors (PbO_2_ and PbMoO_4_), the types and trends of the gas‐phase products in the time dimension were tested (Figure 2d). The results showed that the gas‐phase products mainly consist of propylene, 1,3‐butadiene, CO, and CO_2_. Among them, the content of CO continued to increase overall in the 100 to 600 °C interval and decreased significantly during the 600 °C holding process, suggesting that CO was involved in hypothermic smelting process of zero‐carbon precursors. Combined with the reactions associated with the hypothermic smelting process of zero‐carbon precursors, CO was mainly involved in the reduction reaction of PbO (from PbO_2_ decomposition). In addition, the generation of Pb^0^ also originated from the reduction of lead oxides (PbO_2_ and PbO) by pyrolytic carbon in the LABs polypropylene shells. In addition to the necessary NaOH during the regeneration of Na_2_MoO_4_ from PbMoO_4_, the presence of carbon could greatly reduce the Gibbs free energy values (Δ*G*) of the thermodynamic reactions and promote its spontaneous progression (Figure 2e). Based on Na_2_MoO_4_ and lead compounds (Pb^0^ and PbO) differences in water solubility, A water‐leaching method was developed for the efficient separation of smelting products. XRD results showed that the lead compounds were present in the solid phase product in the form of different crystalline phase PbO_2_,^[^ ^17^ ^]^ while the physical phase of Na_2_MoO_4_ was not detected (Figure 2f). The above results indicate that Na_2_MoO_4_ was successfully transferred into solution. HRTEM images of water‐leaching residues showed that PbO was embedded in biochar in the form of particulate matter (Figure 2g).

### 2.3 Reuse Effectiveness of Regenerated/Residual Na_2_MoO_4_

**Figure 3:**
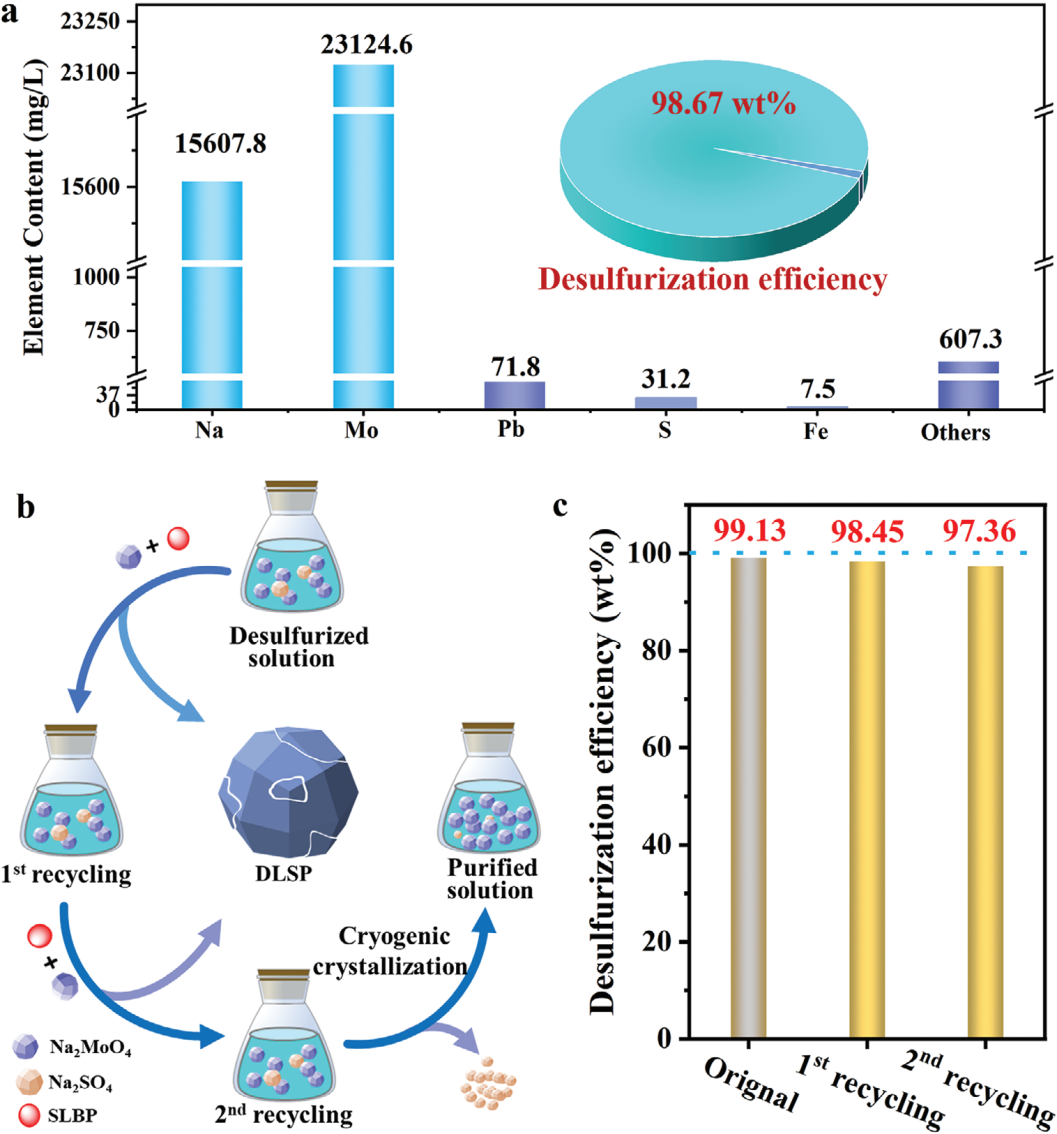
a) Elemental composition and desulfurization efficiency (inset) of water‐leaching liquid. b) Schematic diagram of the second circulation of SLBP desulfurization solution and low‐temperature crystallization purification. c) Circulating desulfurization efficiency of the residual Na_2_MoO_4_ filtrate after the first SLBP desulfurization.

The recovery and reuse of Na_2_MoO_4_ desulfurization is of great importance for reducing the production cost of reclaimed Pb smelting industry.^[^ ^9b^ ^]^ In this study, the recovery of Na_2_MoO_4_ included two main aspects, namely, the recovery of Na_2_MoO_4_ from smelting products by water‐leaching and the reuse of the residual Na_2_MoO_4_ filtrate after the first SLBP desulfurization. As determined by ICP‐OES, the main elemental composition of water‐leaching liquid was Mo and Na (**Figure** **3**a). Interestingly, the desulfurization performance (above 98.67 wt%) of recovered Na_2_MoO_4_ was comparable to that of the fresh Na_2_MoO_4_, which confirmed the feasibility of Na_2_MoO_4_ reuse (inset of Figure 3a). Similarly, to confirm the reuse feasibility and effectiveness of residual Na_2_MoO_4_ in the first SLBP desulfurization solution, two times of recycling (schematic diagram shown in Figure 3b) were carried out to better investigate the changes in the concentration of regenerated Na_2_MoO_4_ and the desulfurization efficiency during the recycling process. The optimal SLBP desulfurization condition (S/S = 2:1, L/S = 5:1, T = 90 °C, and *t* = 4 h) was maintained by adding fresh Na_2_MoO_4_ and deionized water. Figure 3c shows the variation of the desulfurization efficiency of the SLBP during the two cycles. With the increase of cycle numbers, the desulfurization efficiency of SLBP decreased slightly from 98.45 to 97.36 wt%, which could be ascribed to the inhibition of positive desulfurization reaction by the accumulation of a large amount of SO_4_^2−^ in the filtrate. By comparing with desulfurizers reported in the literature, we revealed that recyclable Na_2_MoO_4_ at a leading level in terms of desulfurization efficiency of SLBP (Table S5, Supporting Information).

To remove SO_4_^2−^ from the desulfurization solution after two cycles, Na_2_SO_4_ was separated by low‐temperature crystallization based on the difference in solubility between Na_2_SO_4_ and Na_2_MoO_4_ at low temperatures (Figures S24 and S25, Supporting Information). XRD patterns of the crystalline matters at temperatures ranging from 5 to 15 °C (Figure S26, Supporting Information) reveal that the intensity of characteristic peak for Na_2_MoO_4_^.^2H_2_O remained relatively stable, while the characteristic peak for Na_2_SO_4_ gradually increased with the decreasing reaction temperature. Therefore, the reaction temperature was set at 5 °C. As tested by AAS, the molar ratio of S and Mo in precipitation was 1.04:1, and the amount of residual Na_2_MoO_4_ in the liquid was 13.086 g, with Na_2_MoO_4_ solution containing only 3.9% of S. Although a small amount of Na_2_MoO_4_ was wasted in this way, the higher purity Na_2_MoO_4_ could be used for subsequent efficient desulfurization of SLBP. Noteworthy, 99.83% of Na_2_MoO_4_ could be reused during the circulation of desulfurization liquid according to Equation S5 and Figure S27 (Supporting Information).

### 2.4 Technoeconomic Analysis and Environmental Impact Assessment

**Figure 4:**
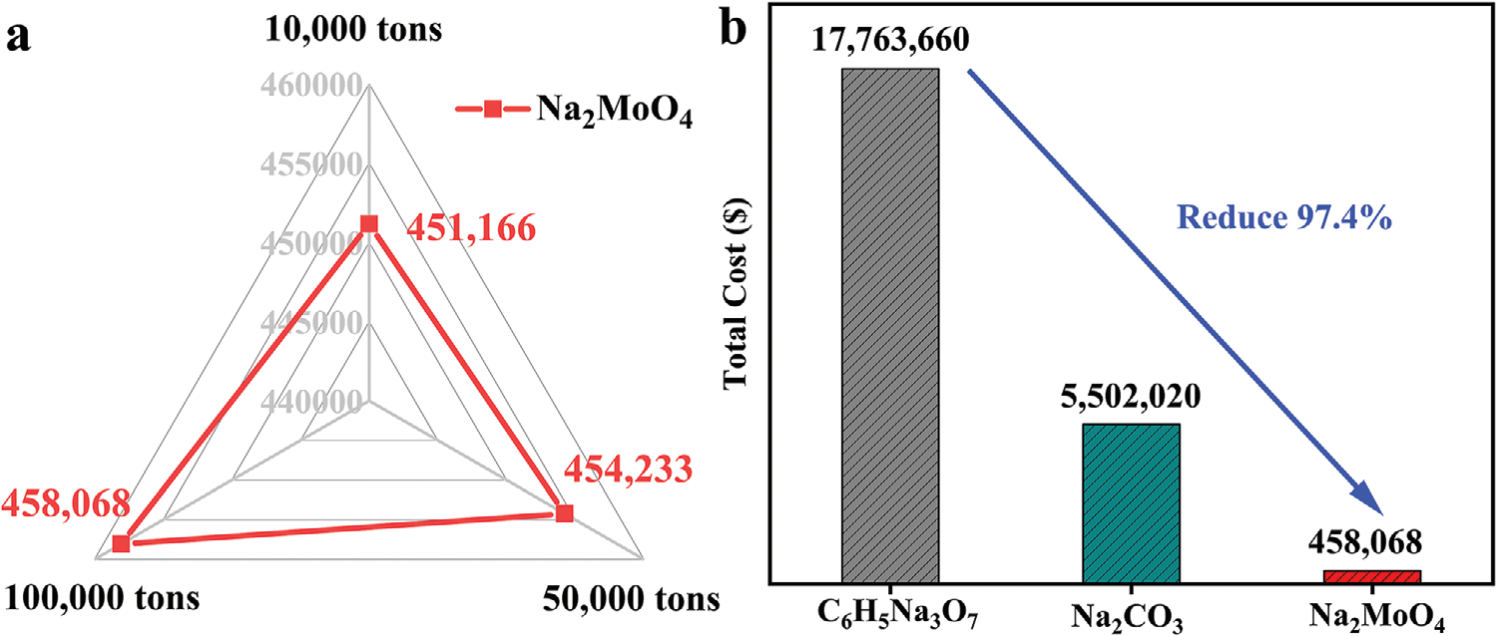
a) Na_2_MoO_4_ and traditional desulfurizers (Na_2_CO_3_ and C_6_H_5_Na_3_O_7_) of disposal costs of 10 000, 50 000, and 100 000 tons of SLBP. b) Total cost of 100 000 tons of SLBP.

The main expense of SLBP recovery was the cost of desulfurizer. Recovering/regenerating Na_2_MoO_4_ desulfurized from SLBP desulfurization solution or smelting products can not only save manufacturing costs but also make an important contribution to the resource recovery and environmental pollution reduction. Here, the cost of Na_2_MoO_4_ desulfurizer used to dispose of 10 000, 50 000, and 100 000 tons of SLBP was first calculated on the basis of 10 000 tons per batch. Based on the proportion of PbSO_4_ in SLBP (≈60 wt%) and the amount and price of the Na_2_MoO_4_ desulfurizer, the cost of 10 000 tons of SLBP treated with Na_2_MoO_4_ could be calculated as 451 166 $. Due to the ability of the system to regenerate 99.83 wt% Na_2_MoO_4_, only a very small number of fresh agents was required to maintain efficient desulfurization of the SLBP in the subsequent processes of 50 000 tons and 100 000 tons. Compared to the cost of disposal of 10 000 tons of SLBP, the cost of treatment of 50 000 tons and 100 000 tons of SLBP increased by 3067 $ and 6903 $, respectively, that is, 454 233 $ and 458 068 $ (**Figure** **4**a). Given the non‐recyclability of traditional desulfurizers, each of the nine subsequent batches of SLBP was considered to be treated at the same cost as the first batch. In contrast, as 99.83 wt% of Na_2_MoO_4_ could be reused in the recovery process of SLBP, the overall cost of treating 100 000 tons with Na_2_MoO_4_ could be even lower, that is, 8.3% and 2.6% of that with the traditional desulfurizers Na_2_CO_3_ (550 202 $) and C_6_H_5_Na_3_O_7_ (1 776 366 $), respectively (Figure 4b). In conclusion, the use of Na_2_MoO_4_ desulfurizer as an alternative to traditional expensive reagents could significantly reduce the costs that long hindered the industrial application of hydrometallurgy technology.

In the context of carbon peak 2030 and carbon neutral 2060,^[^ ^18^ ^]^ this study contributes to CO_2_ emission reduction in the following four aspects, i) The smelting precursors prepared from SLBP can be reduced only in the presence of carbon without the addition of reduced iron powder, ii) Compared with direct pyrometallurgy, the conversion and efficient recovery of Pb phase can be realized at a much lower temperature (600 °C), which greatly reduces the energy consumption of reaction, iii) PbO and Na_2_MoO_4_ desulfurized can be respectively recovered by water‐leaching without the generation of waste liquid and residue as in the traditional process, and the yield of Pb obtained from PbO (95.27 wt%) is significantly higher than that of the conventional process (60–70 wt%), and iv) Up to 99.83 wt% of Na_2_MoO_4_ can be regenerated/recovered for desulfurization of SLBP and the desulfurization efficiency is more than 97.36 wt%, which greatly reduces the industrial production cost and environmental risk.

## 3 Conclusion

In this work, a closed‐loop multi‐step process chain was proposed, consisting of pre‐treatment of LABs, SLBP desulfurization with Na_2_MoO_4_, preparation of smelting precursors, low‐temperature carbon reduction, water leaching, reuse of regenerated/residual Na_2_MoO_4_ and purification of post‐cycle waste salts. Through consumption–regeneration–reuse, the reuse efficiency of Na_2_MoO_4_ was as high as 99.83 wt%. This sustainable Pb recovery process eliminated Pb and SO_2_ pollution and avoided smelting slag generation, which are two major issues for the existing Pb pyrometallurgical process and other developing pyrometallurgical processes. Unlike the traditional hydrometallurgical processes, this new process did not employ any toxic chemicals, such as hydrofluoric acid, and does not release wastewater into the environment. Compared with the traditional smelting technology, the temperature was reduced from >1000 to 600 °C, thus reducing the energy consumption. Such a closed‐loop paradigm could be useful in a broader field of waste management and carbon neutralization, and assisted the sustainable development of new industries in the emergent global energy transition. In short, this study enriched a new variety of the desulfurizers of SLBP, corrected the high loss of lead from the hydrometallurgical process or the pyrometallurgical process alone, supplemented the gap of desulfurized recycling, and then put forward a novel idea of the comprehensive treatment of SLBP.

## 4 Experimental Section

The Experimental Section in the Supporting Information contains detailed information on the chemicals and materials, SLBP desulfurization procedure, preparation of the zero‐carbon precursors (PbO_2_ and PbMoO_4_), synthesis of PbMoO_4_ from the acid purified solution by pH adjustment preparation and water‐leaching separation of smelting products and desulfurization of regenerated Na_2_MoO_4_, reuse and purification procedures of Na_2_MoO_4_ filtrate remaining after the first SLBP desulfurization. Detailed analytical methods and equipment, and DFT calculations were also included.

## Conflict of Interest

The authors declare no conflict of interest.

## Author Contributions

F.L. and N.W.Z. conceived and designed the project. F.L., Y.H.X., W.W.X., X.R.W., and Y.J.C. conducted the experiments. J.J.R., W.W.X., P.X.W., and Z.D. assisted characterizations and experiments. All the authors discussed the results and assisted during the manuscript preparation. N.W.Z. was responsible for the project management.

## Supporting information



Supporting Information

## Data Availability

The data that support the findings of this study are available from the corresponding author upon reasonable request.
