# Supplementary material for: Efficient Desulfurizer Recycling during Spent Lead–Acid Batteries Paste Disposal by Zero‐Carbon Precursor Hypothermic Smelting
Source: Adv Sci (Weinh). 2024 Sep 20;11(43):2405168. doi: 10.1002/advs.202405168 (PMC11578296; doi:10.1002/advs.202405168)
Supplement: Supplementary file 1 — Supporting Information [file ADVS-11-2405168-s001.docx]

Supporting Information

Efficient Desulfurizer Recycling During Spent Lead-Acid Batteries Paste Disposal by Zero-Carbon Precursor Hypothermic Smelting

Fei Li, Neng-Wu Zhu,* Yun-Hao Xi, Wu-Wan Xiong, Ju-Jun Ruan, Xiao-Rong Wei, An-Qi Guo, Yi-Jun Chen, Ping-Xiao Wu, Zhi Dang

F. Li, N.-W. Zhu, Y.-H. Xi, W.-W. Xiong, X.-R. Wei, A.-Q. Guo, Y.-J. Chen, P.-X. Wu, Z. Dang

School of Environment and Energy

South China University of Technology

Guangzhou 510006, China
E-mail: [nwzhu@scut.edu.cn](mailto:nwzhu@scut.edu.cn)

F. Li, J.-J. Ruan

School of Environmental Science and Engineering

Sun Yat-Sen University

Guangzhou 510275, China

N.-W. Zhu, P.-X. Wu, Z. Dang

The Key Lab of Pollution Control and Ecosystem Restoration in Industry Cluster Ministry of Education

South China University of Technology

Guangzhou 510006, China

N.-W. Zhu

Guangdong Environmental Protection Key Laboratory of Solid Waste Treatment and Recycling

South China University of Technology

Guangzhou 510006, China

N.-W. Zhu

Guangdong Provincial Key Laboratory of Solid Wastes Pollution Control and Recycling South China University of Technology

Guangzhou 510006, China

**Experimental Section**

**Chemicals and Reagents**

Spent LABs paste and LABs polypropylene shells were obtained from a local LABs disposal company. Before the experiments, the SLBP and LABs polypropylene shell were dried, crushed and sieved through 200-mesh and 50-mesh sieve respectively. As determined by inductively coupled plasma optical emission spectroscopy (ICP-OES, 93 Agilent ICP 730, USA), the contents of Pb, S, Ca, Mg, Fe, Ba, and Sn were 494397.2 mg/kg, 100563.2 mg/kg, 53151.5 mg/kg, 21281.4 mg/kg, 2505.8 mg/kg, 784.5 mg/kg, and 1068.9 mg/kg, respectively. Ammonium molybdate (Na_2_MoO_4_, purity ≥ 99% w/w), sodium hydroxide (NaOH, purity ≥ 95% w/w), nitric acid (HNO_3_, purity ~ 65% w/w), and ethyl alcohol (CH_3_COOH, purity ~ 99.5% w/w) were provided by Aladdin Chemical Co. Ltd. (Shanghai, China). All of the chemicals were commercially available and used without further purification.

**SLBP Desulfurization Procedure**

All desulfurization experiments were carried out in a 100 mL glass beaker and the reaction temperature was controlled by a thermostatic water bath. The effects of Na_2_MoO_4_/SLBP mass ratio (S/S), liquid-solid ratio (L/S), temperature (T), and reaction time (time) on the treatment of SLBP were studied. Briefly, 10 g of SLBP was added to 50 mL of Na_2_MoO_4_ solution with different S/S (1.5:1, 2:1, 2.5:1, and 3:1). The effect of different L/S ratios (5:1, 6:1, and 7:1) was also investigated under conditions of S/S=2:1, reaction time=4 h, and T=60 °C. Additionally, the effect of different temperatures (30 °C, 60 °C, and 90 °C) and reaction times (2 h, 3 h, and 4 h) was studied to understand their influence on desulfurization. The desulfurization efficiency of the SLBP was calculated as follow:

$$D=\left[ {(m}_{1}\times W_{1}-m_{2}\times W_{2})/{(m}_{1}\times W_{1}) \right]\times100\% (S1)$$

where m_1_ is the mass of SLBP, g; W_1_ is the mass percentage of sulfur of SLBP, wt%; m_2_ is the mass of DLBP, g; and W_2_ is the mass percentage of sulfur of DLBP, wt%.

**Preparation of the Zero-Carbon Precursors (PbO_2_ and Na_2_MoO_4_)**

1 g of DLBP was added to a 100 mL glass beaker containing 80 mL leaching solution with a specified amount of HNO_3_. The slurry was stirred at room temperature (25 ± 1 °C). The effects of the volume ratios of HNO_3_ to water (15:65, 17.5:62.5, and 20:60) and reaction times (1 h, 2 h, and 3 h) on the DLBP were investigated. Under the optimal conditions of nitric purification, the experiment was repeated three times and the filtrate volume fixed at 500 mL. To further recover Pb from the acid purified solution, 80 mL acid purified solution was taken to explore the influence of different pH values (1.64, 3.81, 5.65, 7.78) and reaction times (30 min, 60 min, and 90 min) on Pb yield. During pH adjustment, the recovery efficiency of Pb was calculated as follows:

$$R=\left[ 1-500{(C}_{2}\times V_{2})/80{(V}_{1}\times C_{1}) \right]\times100\% (S2)$$

where V_1_ is the total volume of the leaching solution, mL; C_1_ is the Pb concentration of the leaching solution, mg/L; V_2_ is the volume of the filtrate after reaction, mL; C_2_ is the Pb concentration of the filtrate after reaction, mg/L.

During the whole leaching process, the recovery efficiency of Pb was calculated as follow:

$$R=\left[ 1-500C_{2}\times V_{2}/80{(m}_{2}\times W_{2}) \right]\times100\% (S3)$$

where m_2_ is the mass of DLBP, g; and W_2_ is the mass percentage of Pb in the DLBP, wt%; V_2_ is the volume of the filtrate after reaction, mL; C_2_ is the Pb concentration of the filtrate after reaction, mg/L.

**Preparation and water-leaching separation of smelting products and desulfurization of regenerated Na_2_MoO_4_**

All smelting experiments were carried out in a tube furnace. The effects of reaction temperature, reaction time, and mass of LABs polypropylene shells (carbon source) on the treatment of desulfurization products (PbMoO_4_ and PbO_2_) were studied. Briefly, a certain mass ratio of reactants (PbMoO_4_: PbO_2_: NaOH: LABs polypropylene shells=4.38: 0.66: 2.29: 1.05) was evenly mixed with ethanol and transferred to a magnetic boat to dry at 70 °C (the mass ratio of PbMoO_4_/PbO_2_ was derived from the preparation of the smelting precurdors; the mass of NaOH and LABs polypropylene shells were calculated according to the chemical formula of its desulfurization products). The effect of reaction temperatures (400 °C, 500 °C, 600 °C, 700 °C, and 800 °C) was investigated to study the influence of the smelting on desulfurization products under the conditions of PbMoO_4_: PbO_2_: NaOH: LABs polypropylene shell=4.38:0.66:2.29:1.05 and reaction time=6 h. Further, the effect of different reaction times (6 h, 12 h, and 18 h) and masses of LABs polypropylene shells (2.29 g, 2.79 g, and 3.29 g) were designed to study their influence on smelting. In addition, gases generated during the reduction process of smelting precursors were disposed in an alkali liquor vessel at the end of the reaction equipment (Figure S19). The recovery efficiency of the Pb/Mo was calculated as follow:

$$R=\left[ {(m}_{4}\times W_{4})/{(m}_{3}\times W_{3}) \right]\times100\% (S4)$$

where m_3_ is the mass of the mixture before smelting, g; W_3_ is the mass percentage of Pb/Mo, wt%; m_4_ is the mass of the mixture after smelting, g; and W_4_ is the mass percentage of Pb/Mo, wt%.

10 g of smelting products were added into a 100 mL glass beaker containing 80 mL water. The slurry was stirred at room temperature for 1 h. Lead compounds and Na_2_MoO_4_ solution were recovered by solid-liquid separation. The obtained Na_2_MoO_4_ solution was concentrated to a specific concentration using a rotary evaporator for desulfurization of SLBP under the conditions of S/S=2:1, L/S=5:1, T=90 °C, and t=4 h.

**Reuse and purification procedures of Na_2_MoO_4_ filtrate remaining after the first SLBP desulfurization**

For the residual Na_2_MoO_4_ filtrate after the first SLBP desulfurization, the optimal SLBP desulfurization conditions (S/S=2:1, L/S=5:1, T=90 °C, and t=4 h) were maintained by adding fresh reagents and deionized water. After 4 hours, solids obtained by solid-liquid separation were dried in a vacuum drying oven for 12 h and used for subsequent desulfurization efficiency testing (according to Formula 1). This process was defined as the first cycle. After determining the quality of Na₂MoO₄ in the residual filtrate using ICP-OES, fresh reagents and deionized water were added to the filtrate to repeat the first cycle, which was termed the second cycle, all low temperature crystallization experiments were carried out in a 25 mL glass serum bottle with reaction temperature controlled by an intelligent biochemical incubator. The effects of different temperatures (5 °C, 10 °C, and 15 °C) on the precipitation of Na_2_SO_4_ in the SLBP desulfurization filtrate (10 mL per dose) were studied. After the reaction, the slurry was suctioned to collect filtrate and precipitate separately. The content of S and Mo in the filtrate were detected by ICP-OES. The precipitate was dried by vacuum oven and analysed including phase and elements abundance.

Reuse efficiency of Na_2_MoO_4_ in the loop process of first SLBP desulfurization filtrate was calculated as follow:

$$R=\left[ 1-241.95{(m}_{5}\times W_{5})/95.4 \right]\times100\% (S5)$$

where m_5_ is the mass of the precipitate after low temperature crystallization, g; W_5_ is the mass percentage of Mo, wt%.

Analysis and Equipment

The crystal phases were characterized using X-ray diffraction (XRD, D8 Advance, Bruker, Germany) with Cu Ka radiation, operating at 40 kV and 30 mA at a rate of 2°/min in the 2θ range. The morphologies of the products were characterized by field emission scanning electron microscopy (FE-SEM, Quanta650, Hitachi, USA), and energy-dispersive spectroscopy (EDS, TM3030, Hitachi, USA). Fourier transformed infrared (FTIR) spectra of the samples were analysed by a VERTEX-70 spectrometer, and KBr was used as a blank control. The contents of main elements in the samples were determined by ICP-OES (Agilent ICP 730, USA) or AAS (AA6880, Shimadzu, Japan). The anion contents of the DLBP filtrate were determined by anion chromatography (Thermo Fisher ICS-1500, USA). The Gibbs free energy values (ΔG) of the possible thermodynamic reactions during the whole desulfurization process were calculated by HSC Chemistry 6.0. The pH of the solutions was measured with a pH meter (PHS-3E, INESA Scientific Instrument Co. Ltd. China).

**Sample Digestion Method**

1. 0.5g samples was added to a 50 mL polytetrafluoroethylene digestion tank;

2. Different acids including HNO_3_, HClO_4_, and HF were added into the polytetrafluoroethylene digestion tank in sequence, and the ratio of different acids was 10 mL of HNO₃, 5 mL of HClO₄, and 10 mL of HF (2:1:2);

3. Cover the polytetrafluoroethylene digestion tank with a lid and place it in the digester, and set the ramp-up time to 30 min, followed hold at 200 ℃ for 240 min;

4. After digestion, solution became white and transparent or light yellow, indicating the digestion process was successful. Otherwise, different acids were added in the same ratios and heated at 200 ℃ for digestion until the residual solution turned transparent solution.

5. Finally, the residual solution was cooled to room temperature by nature cooling and transferred to a 25 mL volumetric flask. The metal content in diluted solution was determined by AAS/ICP-OES.

**DFT Calculations**

All calculations were performed in the framework of the density functional theory with the B3LYP method. The 6-311g(d,p) basis set was used for S, O, Na and Ca atoms and the LANL2DZ basis set was used for Mo and Pb atoms. Long range van der Waals interaction were described by the DFT-D3 approach.^[1]^ The optimal structure was calculated using the PCM solvent model in aqueous solution.

**
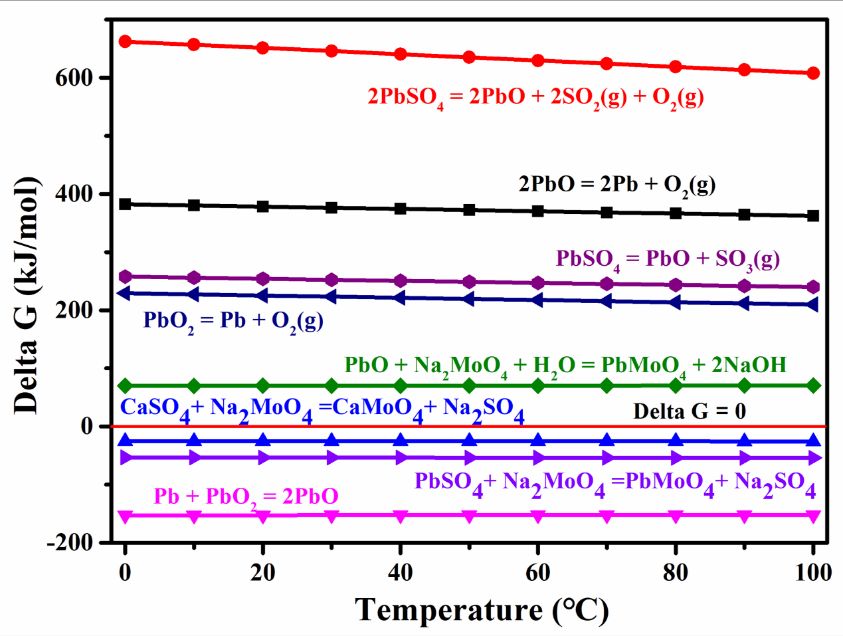
**

**Figure S1.** Gibbs free energy values (ΔG) of possible thermodynamic reactions during the desulfurization process of SLBP.


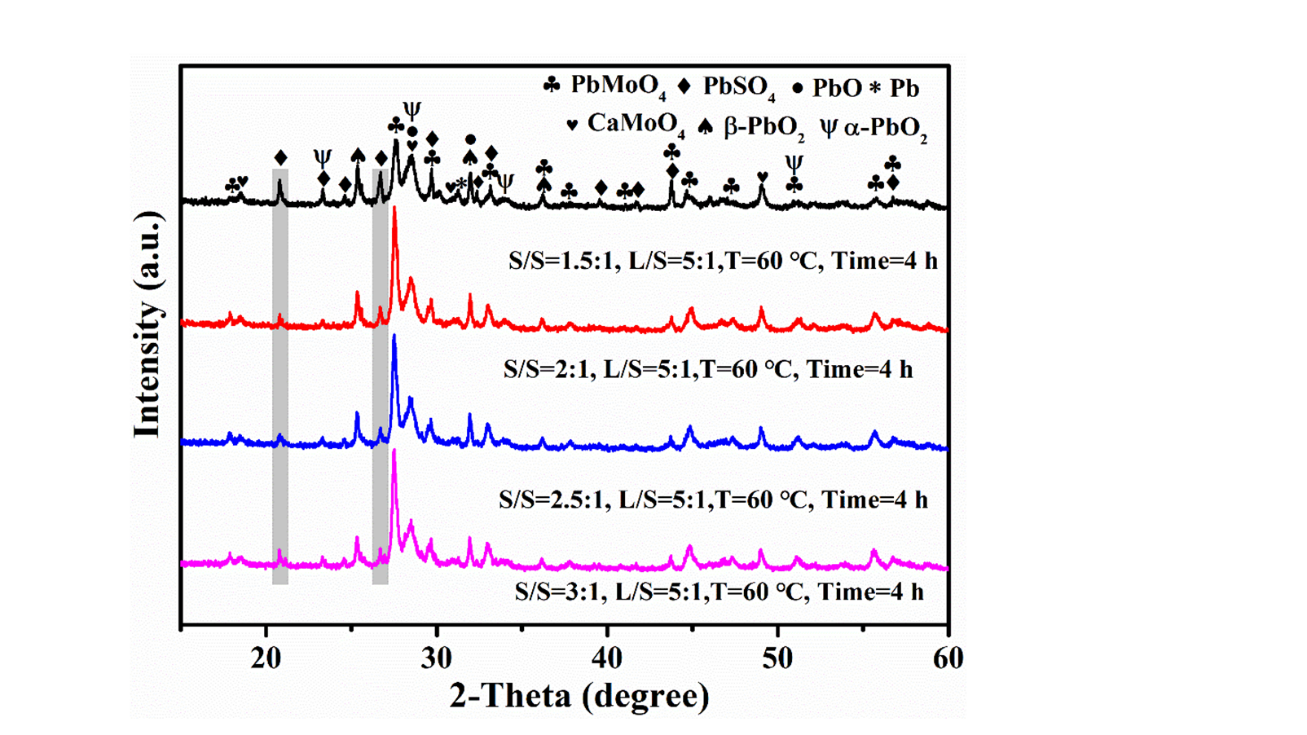


**Figure S2.** Effect of Na_2_MoO_4_/SLBP mass ratio (S/S) on the desulfurization of SLBP.


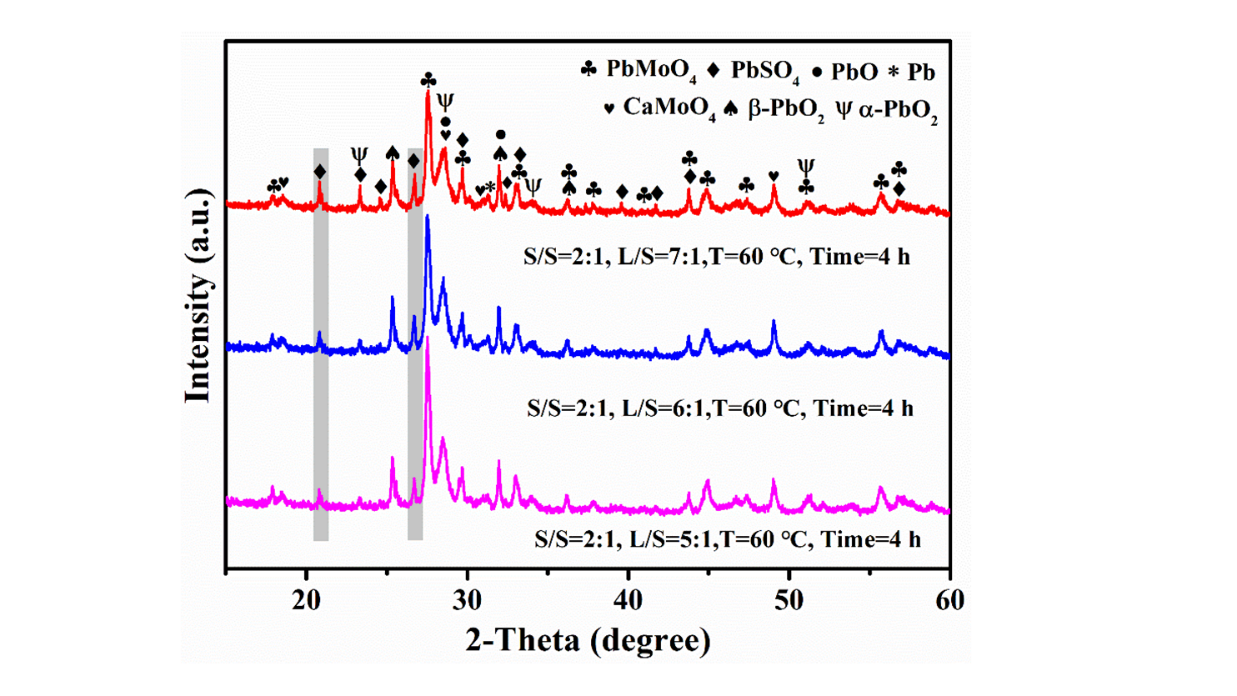


**Figure S3.** Effect of liquid-solid ratio (L/S) on the desulfurization of SLBP.


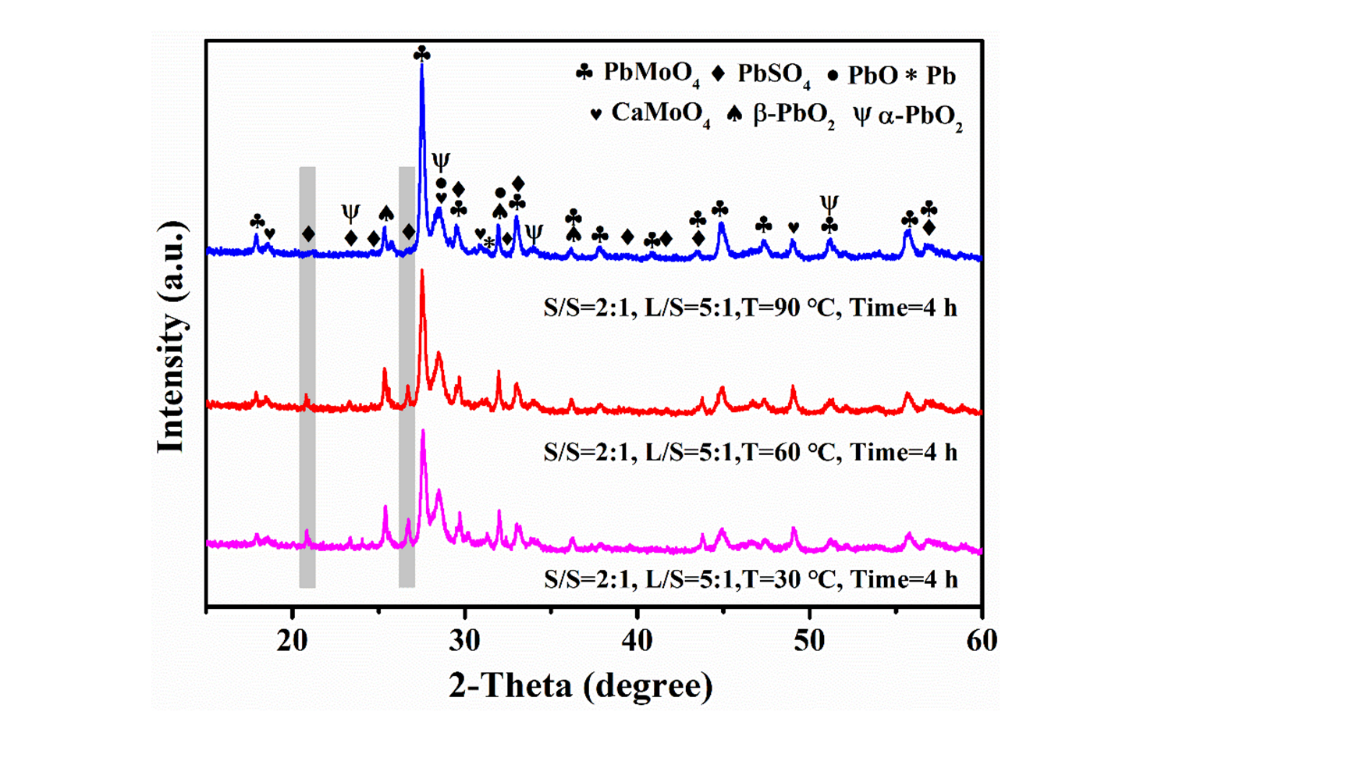


**Figure S4.** Effect of the reaction temperature (T) on the desulfurization of SLBP.


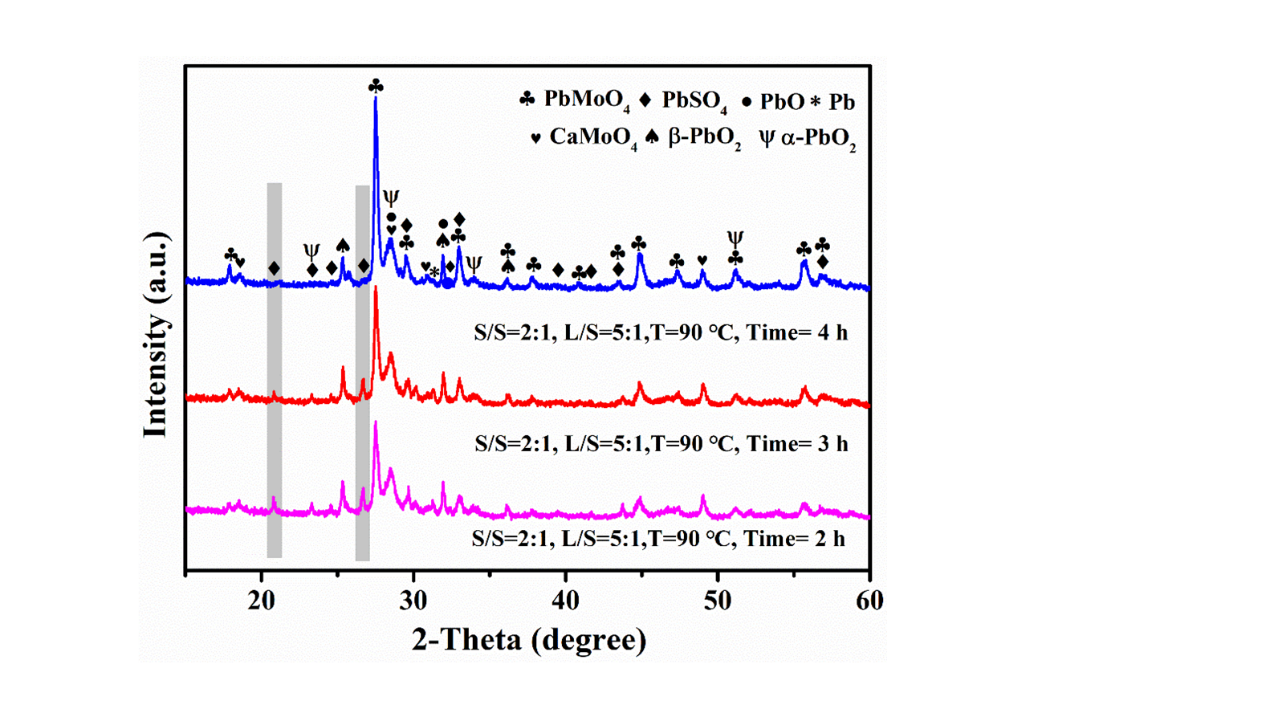


**Figure S5.** Effect of reaction time (t) on the desulfurization of SLBP.


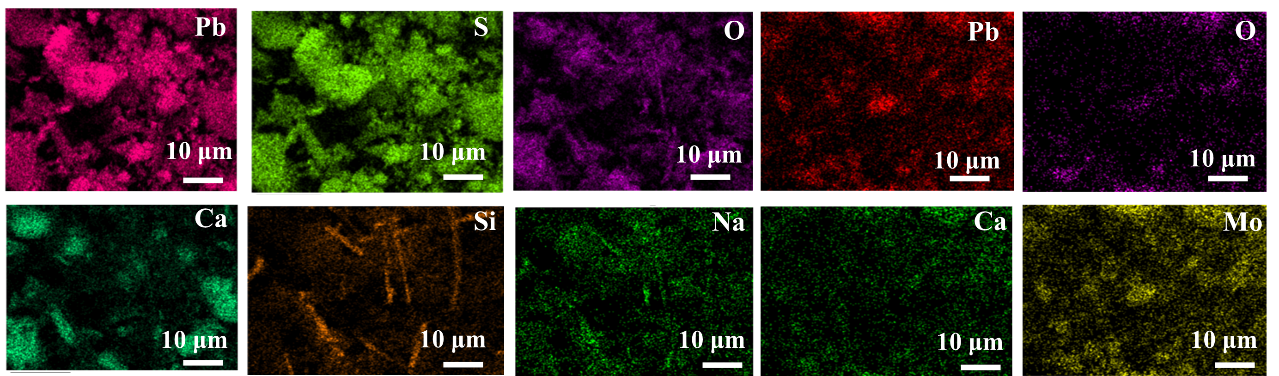


**Figure S6.** Elemental mapping images of SLBP (Top row) and DLBP (Bottom row).


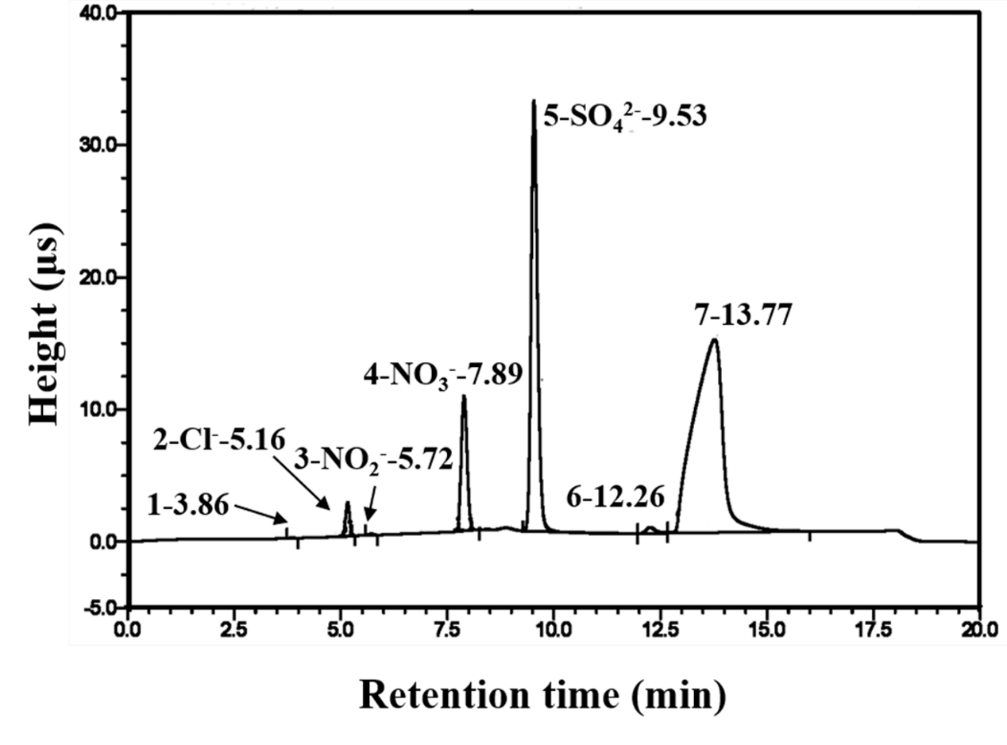


**Figure S7.** Anion chromatography of DLBP filtrate.


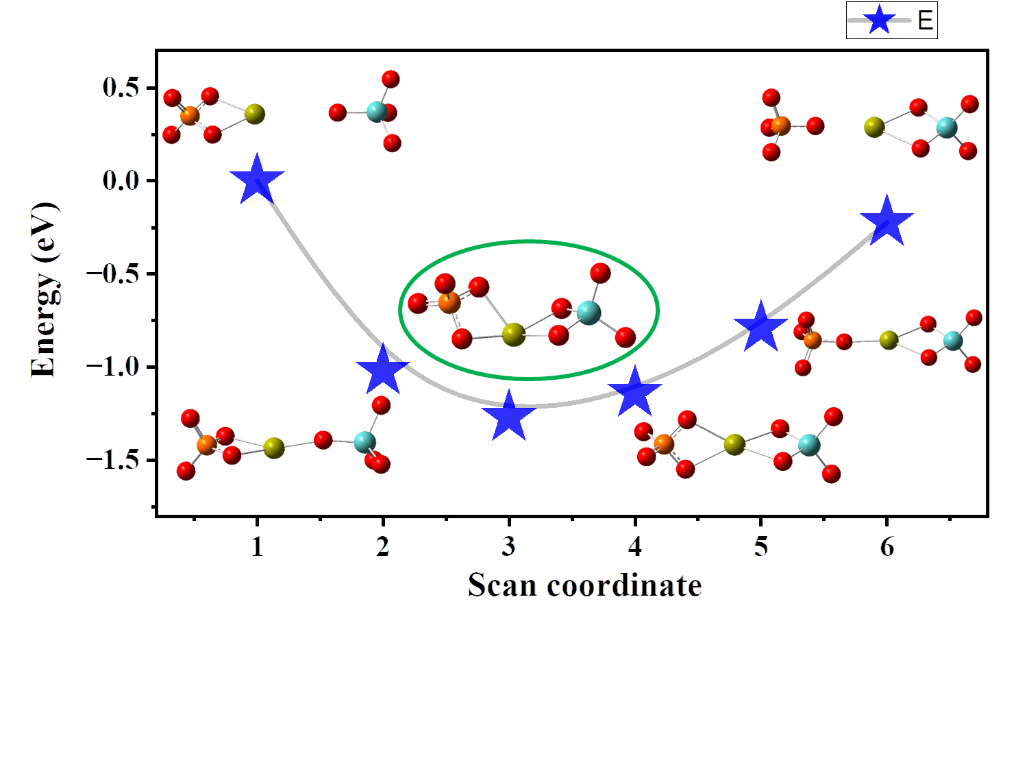


**Figure S8.** Energetic and structural changes in the removal of SO_4_^2-^ from CaSO_4_^.^2H_2_O in the presence of Na_2_MoO_4_.


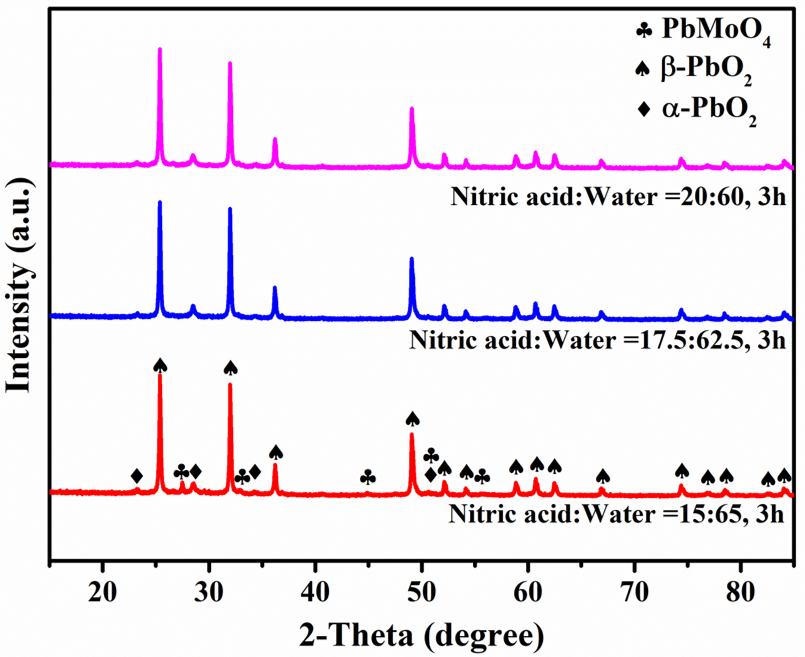


**Figure S9.** Effect of HNO_3_/H_2_O volume ratio on the DLBP.


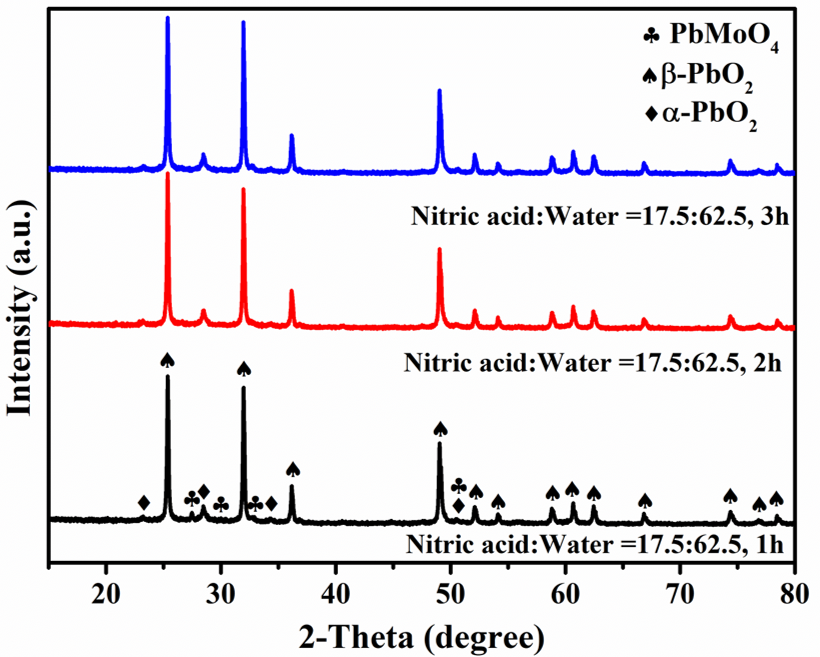


**Figure S10.** Effect of reaction time (t) on the DLBP.


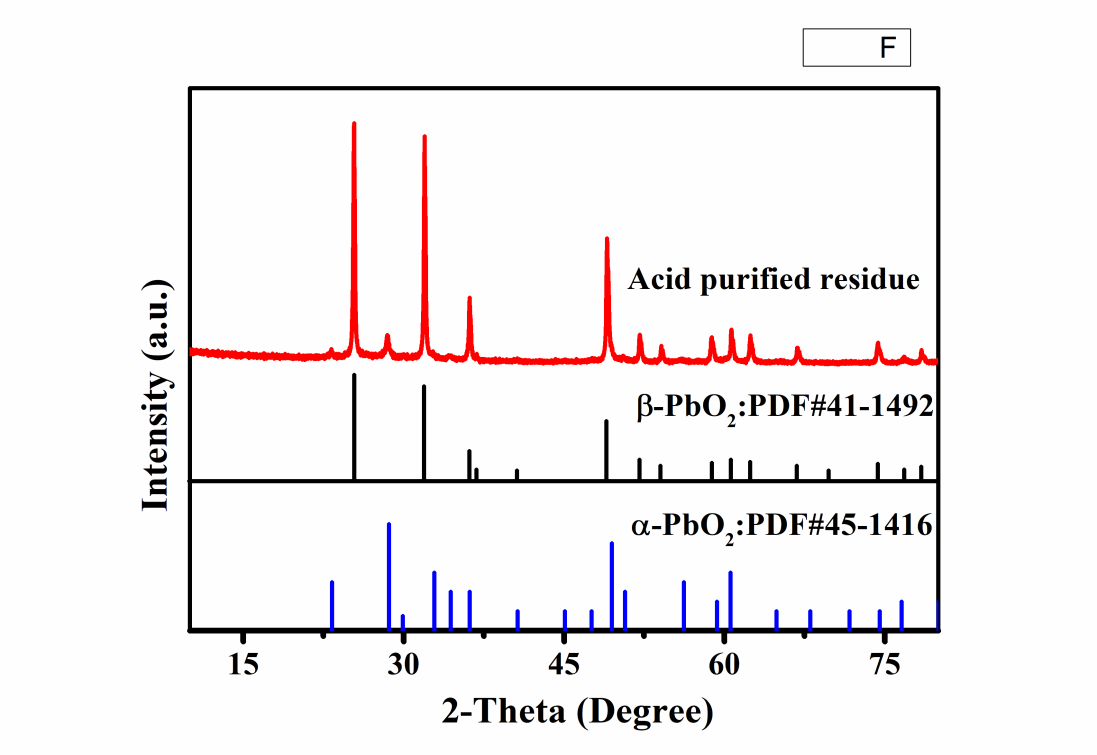


**Figure S11.** XRD of acid purified residue (HNO_3_/H_2_O volume ratio = 17.5:62.5 and t=2 h).


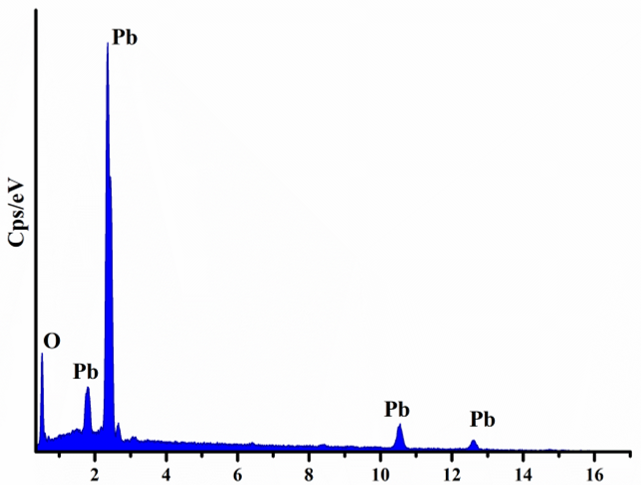


**Figure S12.** EDS of HNO_3_ purified residues.


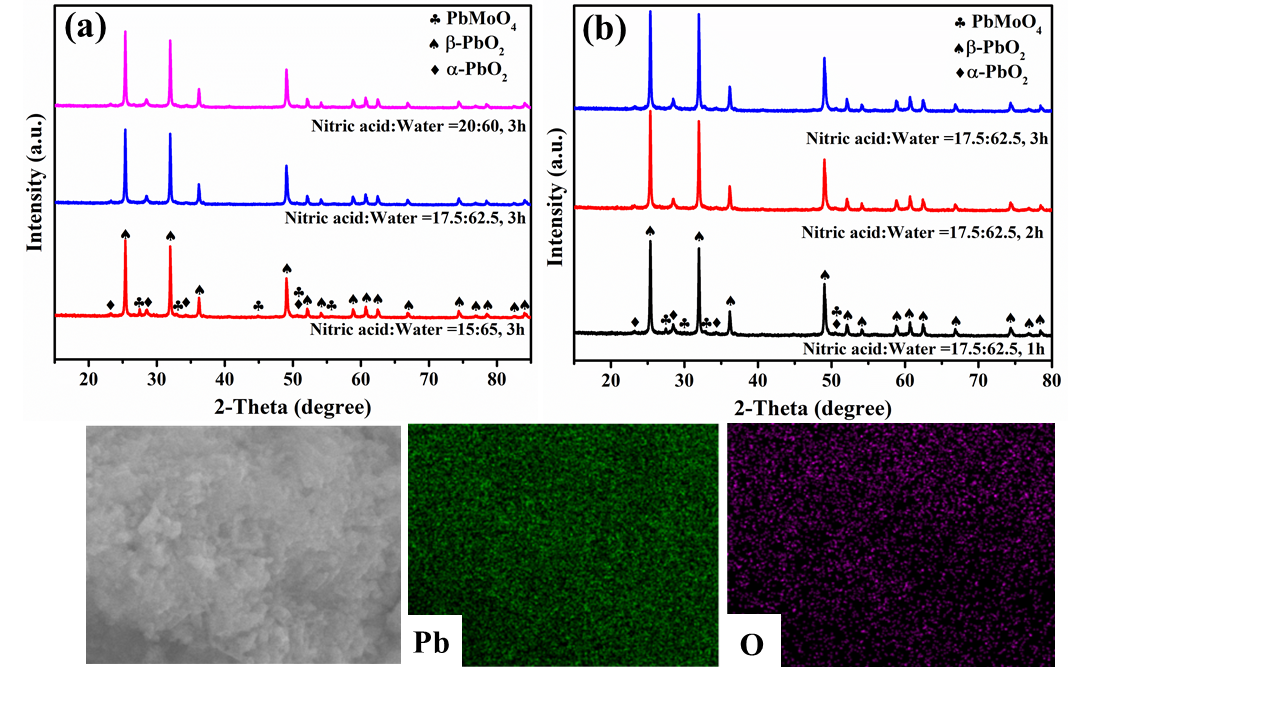


**Figure S13.** Elemental mapping images of HNO_3_ leaching residues.

**

**

**Figure S14.** XRD patterns of re-crystallization product products of HNO_3_ purified solution under different pH values.


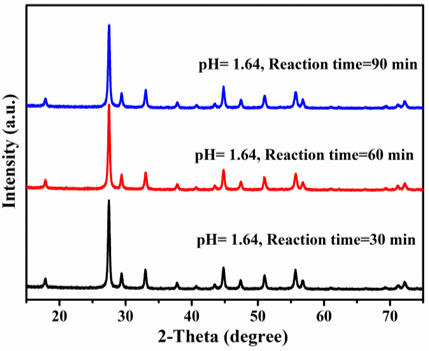


**Figure S15.** Effect of different reaction times in the HNO_3_ purified solution under pH=1.64.


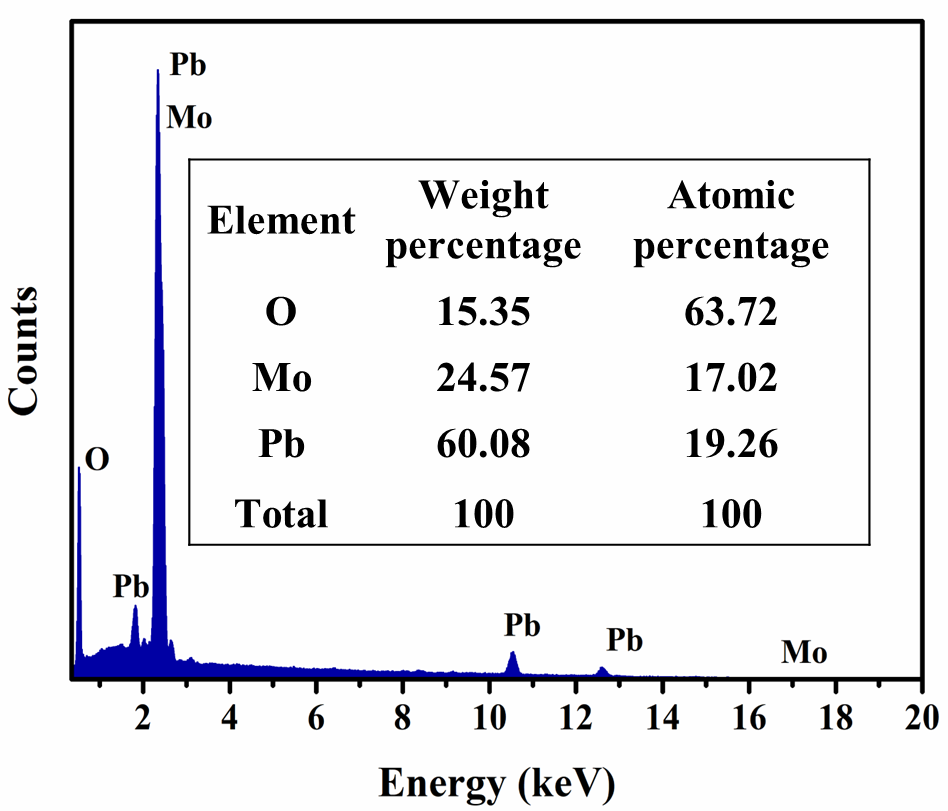


**Figure S16.** EDS images of the re-crystallization product of HNO_3_ purified solution (pH = 1.64 and reaction time=90 min).


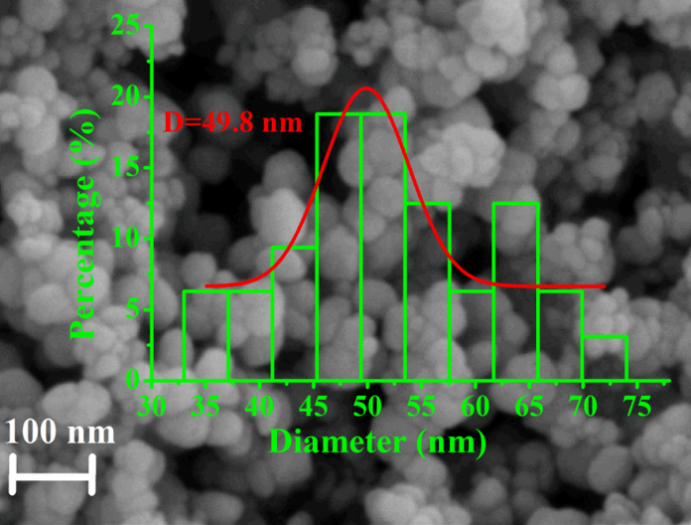


**Figure S17.** SEM image and average particle size of PbMoO_4_.


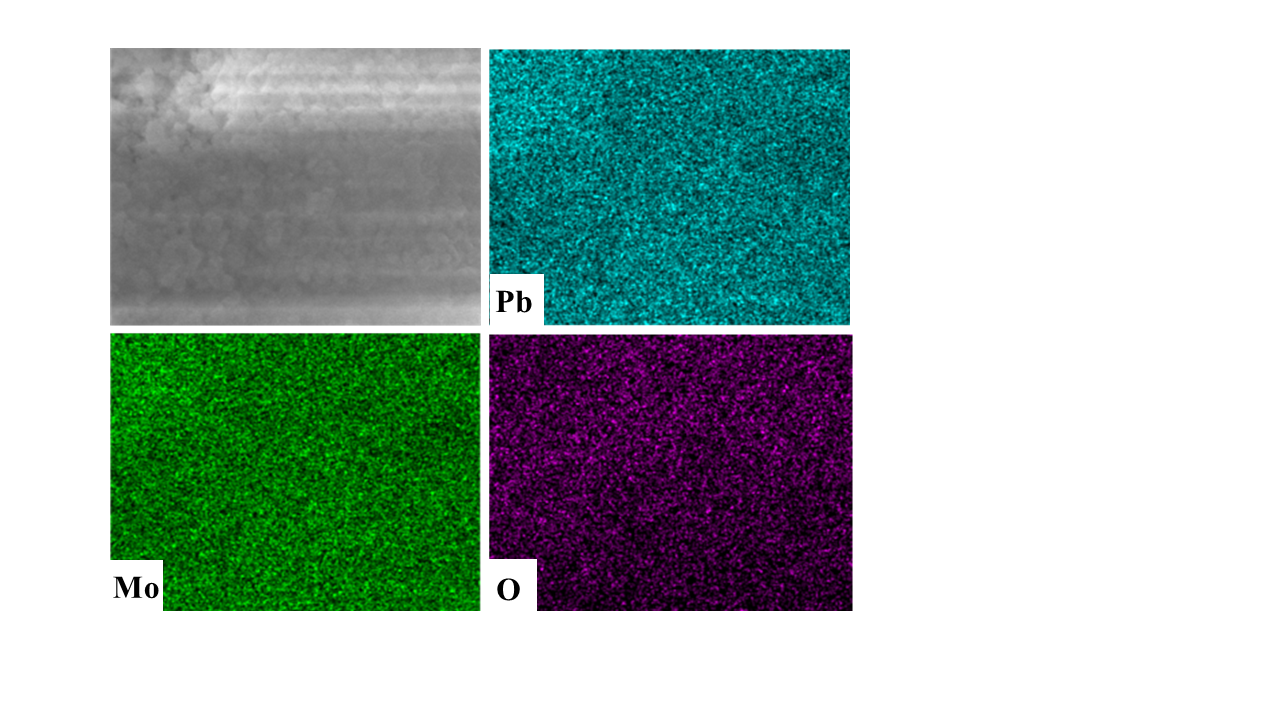


**Figure S18.** Elemental mapping images of re-crystallization products (pH=1.64 and reaction time=90 min).


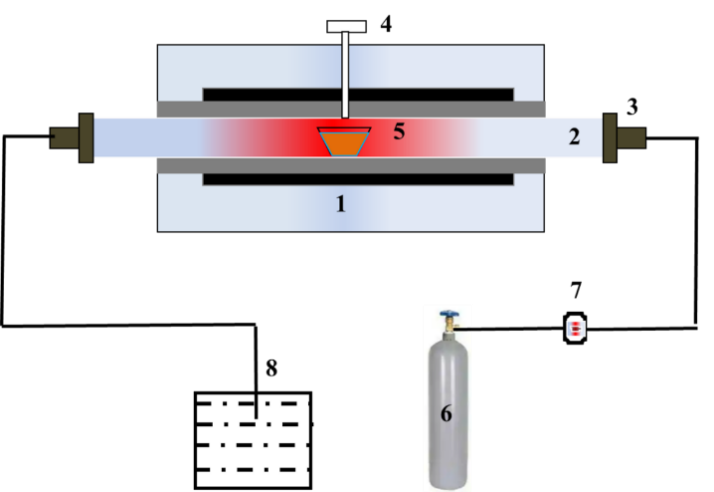


**Figure S19.** Schematic of the experimental apparatus for reduction of smelting precursors, (1) Horizontal tube furnace; (2) Alumina work tube; (3) Sealing flange; (4) Pt-PtRh thermocouple; (5) Alumina crucible; (6) N_2_ gas cylinder; (7) Flowmeter; (8) Alkali liquor vessel.





**Figure S20.** Effect of the reaction temperature (T) on the smelting precursor phases.





**Figure S21.** Effect of reaction time (t) on the smelting precursor phases.





**Figure S22.** Effect of different LABs polypropylene shells doses on the smelting precursor phases.


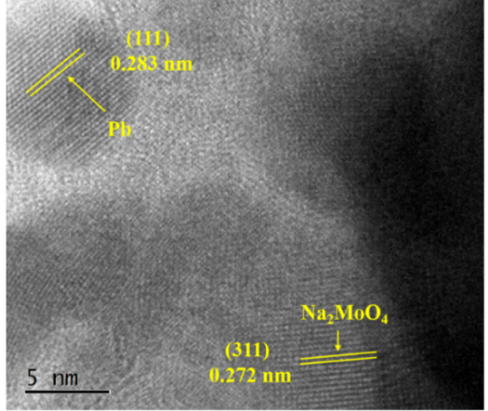


**Figure S23.** HRTEM images of smelting products


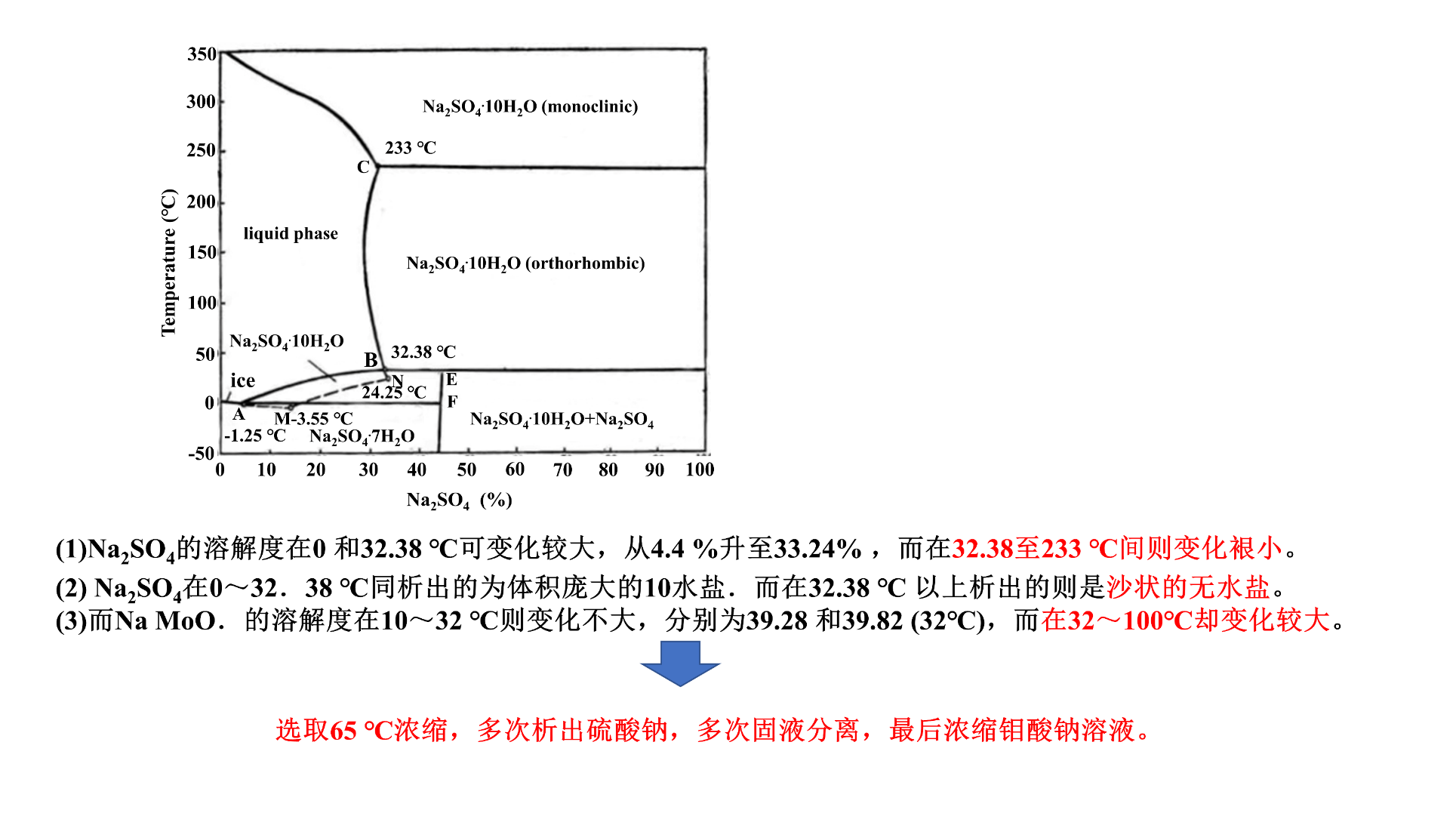


**Figure S24.** System of Na_2_SO_4_-H_2_O (Lange’s Handbook of Chemistry).


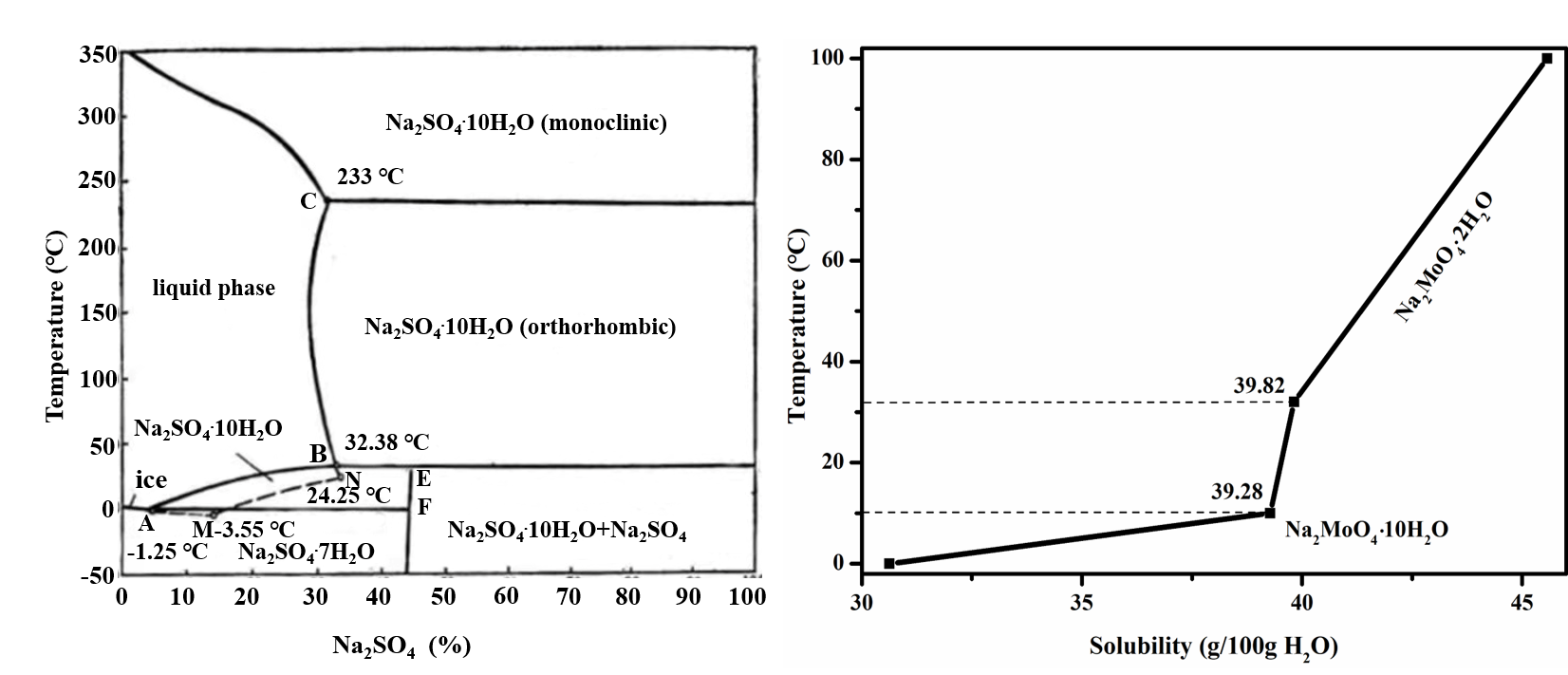


**Figure S25.** Solubility of Na_2_MoO_4_ in water.


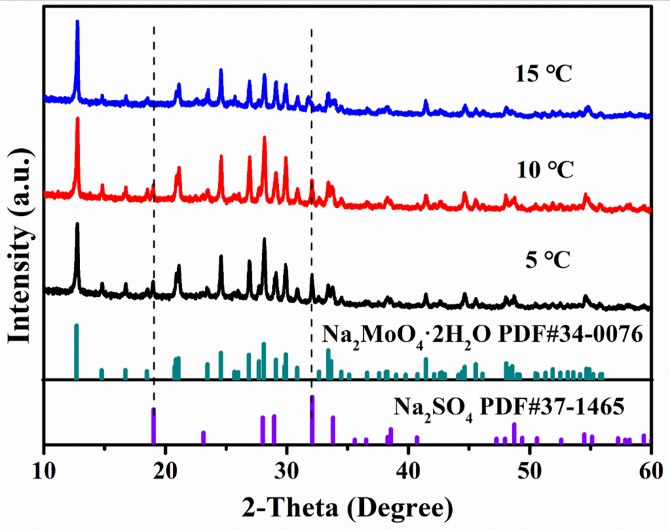


**Figure S26.** XRD patterns of precipitated solid at different temperatures after 2 cycles of desulfurization solution.


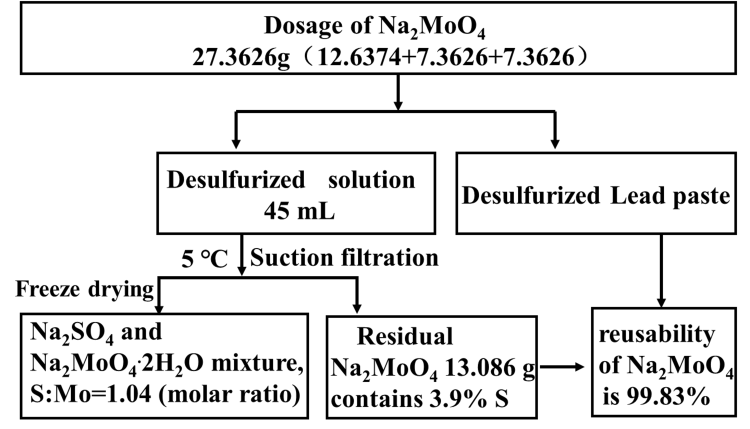


**Figure S27.** Material balance of Na_2_MoO_4_ after 2 cycles desulfurization solution.

**Table S1.** Main element content of obtained DLBP.

| Composition | Pb | Mo | S | Ca | Fe | Ba | Sn |
| --- | --- | --- | --- | --- | --- | --- | --- |
| Content [mg/kg] | 449086.7 | 254129.8 | 1355.4 | 50573.0 | 2219.6 | 689.0 | 1149.4 |

**Table S2.** The types and contents of anion in the filtrate of the SLBP (S/S=2:1, L/S=5:1, T=90 °C, and t=4 h)

| No. | Ret.time  [min] | Peak name | Height  [μs] | Area  [μs*min] | Rel.Area [%] | Amount  [mg/L] |
| --- | --- | --- | --- | --- | --- | --- |
| 1 | 3.86 | n.a. | 0.065 | 0.007 | 0.03 | n.a. |
| 2 | 5.16 | Cl^-^ | 2.541 | 0.282 | 1.46 | 41.587 |
| 3 | 5.72 | NO_2_^-^ | 0.090 | 0.011 | 0.06 | 2.410 |
| 4 | 7.89 | NO_3_^-^ | 10.279 | 1.633 | 8.48 | 440.820 |
| 5 | 9.53 | SO_4_^2-^ | 32.563 | 5.651 | 29.33 | 1245.960 |
| 6 | 12.26 | n.a. | 0.474 | 0.129 | 0.67 | n.a. |
| 7 | 13.77 | n.a. | 14.581 | 11.556 | 59.97 | n.a. |

**Table S3.** Concentrations of Pb and impurities in the lead nitrate solutions [mg/L].

| Pb | Ca | Mg | Al | Cu | Zn | Ba | Fe | S | Sn |
| --- | --- | --- | --- | --- | --- | --- | --- | --- | --- |
| 1128.5 | 277.4 | 122.9 | 4.243 | 2.616 | 1.06 | 3.038 | 11.10 | 81.77 | 1.065 |

**Table S4.** The content of lead in the leaching solution with different reaction time under the condition of pH = 1.64.

| Time [min] | 0 | 30 | 60 | 90 |
| --- | --- | --- | --- | --- |
| Content [mg/L] | 2021.1±9.8 | 48.956±1.2 | 15.796±0.14 | 0.689±0.07 |

**Table S5.** Efficiency comparison of different desulfurization technologies.

| Desulfurization Method | Desulfurization Efficiency [%] | Reusability | Ref. |
| --- | --- | --- | --- |
| CH_3_COOH-Na_3_C_6_H_5_O_7_ | 99.1 | No | [2] |
| C_4_H_6_O_6_-C_4_H_4_Na_2_O_6_ | 99.51 | No | [3] |
| NaOH | 98.95 | No | [4] |
| NaOH | 99.4 | No | [5] |
| Na_2_CO_3_ | 96.3 | No | [6] |
| (NH_4_)_2_CO_3_ | 98.4 | No | [7] |
| Na_2_MoO_4_ | 99.13 | Yes | This work |

**References**

[1] S. Grimme, J. Antony, S. Ehrlich, H. Krieg, *Journal of Chemical Physics* **2010**, *132*, 154104.

[2] X. Zhu, X. He, J. Yang, L. Gao, J. Liu, D. Yang, X. Sun, W. Zhang, Q. Wang, R. V. Kumar, *J. Hazard. Mater*. **2013**, *250-251*, 387.

[3] L. Ye, L. Duan, W. Liu, Y. Hu, Z. Ouyang, S. Yang, Z. Xia, *Hydrometallurgy* **2020**, *197*, 105450.

[4] X. Deng, W. Liu, D. Zhang, L. Chen, Z. Liu, T. Yang, *Sep. Purif. Technol.* **2021**, *259*, 118115.

[5] J. Pan, X. Zhang, Y. Sun, S. Song, W. Li, P. Wan, *Ind. Eng. Chem. Res*. **2016**, *55*, 2059.

[6] W. Yu, P. Zhang, J. Yang, M. Li, Y. Hu, S. Liang, J. Wang, S. Li, K. Xiao, H. Hou, J.

Hu, R. V. Kumar, *J. Clean. Prod*. **2019**, *210*, 1534.

[7] X. Zhu, L. Li, X. Sun, D. Yang, L. Gao, J. Liu, R. V. Kumar, J. Yang, *Hydrometallurgy*

**2012**, *117-118*, 24.
